# SUMOylated Golgin45 associates with PML-NB to transcriptionally regulate lipid metabolism genes during heat shock stress

**DOI:** 10.1038/s42003-024-06232-3

**Published:** 2024-05-06

**Authors:** Shuaiyang Jing, Jingkai Gao, Neeraj Tiwari, Yulei Du, Lianhui Zhu, Bopil Gim, Yi Qian, Xihua Yue, Intaek Lee

**Affiliations:** 1 School of Life Science and Technology, ShanghaiTech University, Shanghai, China; 2 University of Chinese Academy of Sciences, Beijing, China; 3 Department of Cell Biology, Yale University School of Medicine, New Haven, CT 06520 USA; 4 School of Physical Science and Technology, ShanghaiTech University, Shanghai, China

**Keywords:** Golgi, Golgi

## Abstract

Golgin tethers are known to mediate vesicular transport in the secretory pathway, whereas it is relatively unknown whether they may mediate cellular stress response within the cell. Here, we describe a cellular stress response during heat shock stress via SUMOylation of a Golgin tether, Golgin45. We found that Golgin45 is a SUMOylated Golgin via SUMO1 under steady state condition. Upon heat shock stress, the Golgin enters the nucleus by interacting with Importin-β2 and gets further modified by SUMO3. Importantly, SUMOylated Golgin45 appears to interact with PML and SUMO-deficient Golgin45 mutant functions as a dominant negative for PML-NB formation during heat shock stress, suppressing transcription of lipid metabolism genes. These results indicate that Golgin45 may play a role in heat stress response by transcriptional regulation of lipid metabolism genes in SUMOylation-dependent fashion.

## Abstract (web summary)

Golgin45, a Golgi structural protein, is heavily SUMOylated and heat shock stress increases Golgin45 SUMOylation modified by SUMO3 and promotes its inclusion into PML-NBs to transcriptionally regulate lipid metabolism genes.

## Introduction

Small ubiquitin-like modifier (SUMO) is a well-known post-translational modification that is covalently attached to lysine residues of hundreds of target proteins^1^. Unlike ubiquitination, SUMO modification likely occurs at SUMO consensus sequence, ΨKxE (Ψ= hydrophobic residue, typically L/I/V, x = any amino acid residue), although it is also known to be attached to non-consensus sites under stress condition^2–4^. There are additional sumoylation motifs reported by unbiased proteomic analysis, for examples, inverted SUMOylation motif (E/DXKΨ) and hydrophobic cluster SUMOylation motif (ΨΨΨKXE)^5,6^. SUMO modification also accurs via interaction with a SUMO-interacting motif (SIM). SUMO targets that require a SIM for modification are frequently sumoylated on multiple sites, including lysine residues that are not part of consensus motifs^7^.

In mammals, five SUMO isoforms have been identified to date^8^. A number of studies have shown that these SUMO isoforms play different roles in various physiological contexts, and there is also high functional redundancy, which had made it difficult to study their specific functions^1,6,8^. While the most extensively studied SUMO isoform is SUMO1, SUMO1 is largely dispensable for mouse embryonic development, whereas SUMO2/3 play more crucial roles^9^. SUMOylation has been shown to modulate protein-protein interaction, intracellular localization of proteins, enzymatic activity, immune cell functions, cellular stress response and inflammatory responses, etc^6^.

One of the well characterized roles for protein SUMOylation is regulation of promyelocytic leukemia nuclear bodies (PML-NBs) formation, which undergoes dynamic regulation by protein SUMOylation^10–12^. PML-NBs are involved in various genome maintenance pathway and cellular stress response, including the DNA damage response, innate immunity during viral infection, DNA repair, telomere homeostasis, cellular senescence and p53-associated apoptosis^13–16^.

We have recently reported that Golgin45, a Golgi structural protein, functions as a major docking factor for Tankyrase-1 (TNKS1) recruitment to Golgi membranes, and that TNKS1-dependent poly (ADP-ribosyl)ation (PARylation) of Golgin45 regulates Golgin45 protein level via proteasomal degradation in interphase cells, leading to modulation of intracellular trafficking dynamics of Golgi-resident glycosyltransferases^17^. These results revealed a novel regulatory mechanism that influences Golgi structure and function via PARylation of its structural protein. Yet, exactly how TNKS1-dependent Golgin45 PARylation is controlled had remained elusive.

Studies have suggested that ubiquitination, PARylation and SUMOylation influence one another in various physiological and pathological conditions (reviewed in ref. ^18^). To further investigate the regulatory mechanism behind TNKS1-dependent Golgin45 PARylation, we first posited that Golgin45 may be subjected to both PARylation and SUMOylation and the interplay between them might dictate Golgin45 protein stability during interphase.

Our results show that Golgin45 is a heavily SUMOylated protein with at least 8 SUMOylation sites primarily in its N-terminal domain and its SUMOylation is facilitated by Importin-β2-mediated translocation into the nucleus. Importantly, Golgin45 SUMOylation greatly increased upon heat shock stress. Whereas Golgin45 is mostly modified by SUMO1 under steady state condition, SUMO2/3 conjugation was greatly increased under heat shock stress, facilitating its inclusion into PML-NBs. Furthermore, expression of SUMOylation deficient Golgin45 mutants inhibited the formation of PML-NBs and suppressed transcription of lipid metabolism genes.

Taken together, these results strongly suggest that SUMOylated Golgin45 may play an important role in heat shock stress response by transcriptional regulation of lipid metabolism genes.

## Results

### Golgin45 is a SUMOylated protein

To test whether Golgin45 can be modified by SUMO, we transiently transfected HeLa cells with mCherry-Golgin45, FLAG-Ubc9 WT (a SUMO E2 conjugase) or dominant negative mutant (DN) and myc-SUMO1 overnight, followed by treatment with or without TAK981 (SUMO E1 inhibitor) for 12 h, in order to directly test SUMO1 conjugation to Golgin45. Cells were then lysed in RIPA buffer and subjected to western blots for changes in Golgin45 protein.

**Fig. 1:**
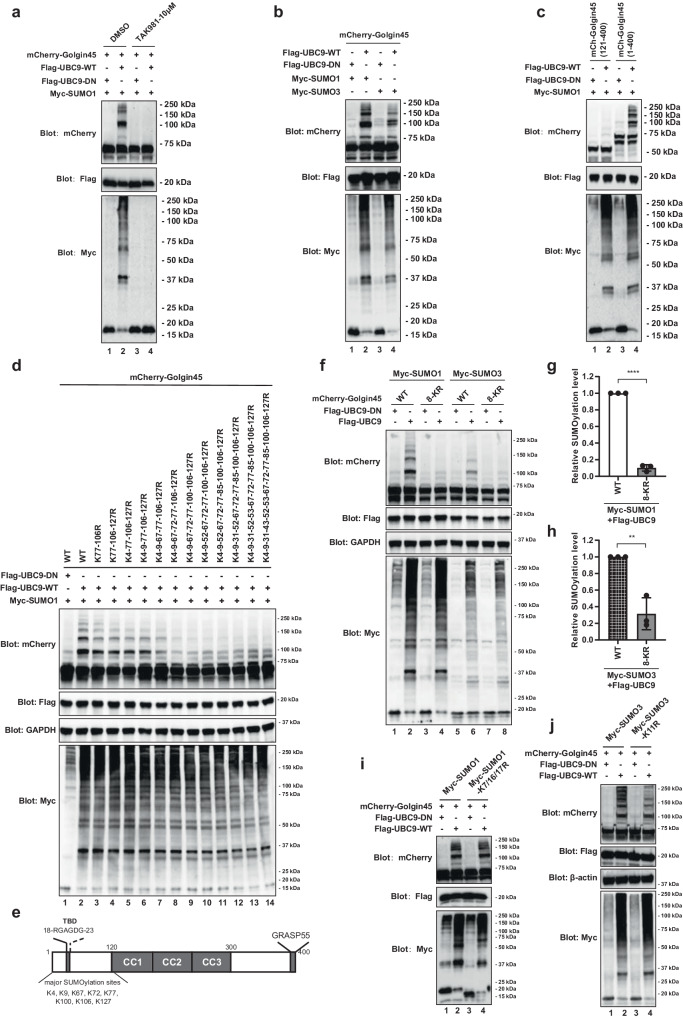
Golgin45 is subjected to SUMO1/3 modification. **a** Golgin45 is robustly conjugated to SUMO1 and the SUMOylation of Golgin45 is abolished by a SUMO-activating enzyme (SAE) inhibitor TAK981. Hela cells were transiently transfected with the indicated plasmids overnight, then the cells were treated with DMSO or 10 μM TAK981 for 12 h and then SUMO1-modified Golgin45 was analyzed by western blot. **b** Golgin45 is SUMOylated by both SUMO1 and SUMO3. Hela cells were transfected with myc-SUMO1/3, Flag-UBC9-WT or its dominant-negative (DN) mutation and mCherry-Golgin45 for 18 h, then the cells were lysed and SUMOylation of Golgin45 was examined by western blot. **c** The SUMOylation sites modified by SUMO1 lie within the N terminal of Golgin45. Hela cells were transfected with mCherry-Golgin45 (121–400) (N terminal deleted truncation) or mCherry-Golgin45 (1–400) (full length) with myc-SUMO1 and Flag-UBC9 for 18 h, the mCherry-Golgin45 SUMOylation was analyzed by western blot. **d** Golgin45 is SUMOylated at multiple sites. Hela cells were transfected with the indicated plasmids for 18 h. Cells were lysed and analyzed by western blotting using anti-mCherry, anti-Flag and anti-Myc. **e** Schematic of Golgin45 domains, showing the identified SUMO acceptor sites. TBD Tankyrase binding domain, CC Coiled-coil domain. **f**–**h** Mutation of the 8 SUMOylation sites (8-KR) reduces the Golgin45 SUMOylation modified by SUMO1 or SUMO3. HeLa cells were transfected with mCherry-Golgin45 (WT or 8-KR) and Flag-UBC9 (WT or DN) and analyzed by western blot (**f**). Relative levels of SUMO1 (**g**) or SUMO3 (**h**) conjugated mCherry-Golgin45 are presented as mean ± SD. Statistical analysis of relative SUMOylation was performed by one-way ANOVA with Tukey’s multiple comparisons. *n* = 3 independent experiments. **P < 0.01.****P < 0.0001. **i**, **j** Hela cells were transfected with the indicated plasmids for 18 h. Cells were lysed and analyzed by western blotting. Representative blots are shown and experiments were repeated three times.

The results showed multiple bands of mCherry-Golgin45 above the typical ~75 kDa band, indicating that Golgin45 is likely to be SUMOylated at multiple sites (Fig. 1a; compare lane 1 and 2). In addition, the observed upper bands of Golgin45 were greatly reduced by treatment with TAK981 (Fig. 1a; compare lane 2 and 4), confirming the finding that Golgin45 is indeed a SUMOylated protein.

To explore how Golgin45 SUMOylation may be regulated, cells were similarly transfected with mCherry-Golgin45, FLAG-Ubc9 WT (or dominant negative) and myc-SUMO1 overnight, followed by glucose starvation, Torin1 (inducer of autophagy), Doxorubicin (DOXO, DNA damage inducer), TAK243 (ubiquitin E1 inhibitor), TAK981 and tert-Butylhydroquinone (TBHQ, oxidative stress) for 12 h (Supplementary Fig. 1). Other than Torin1 and TAK981 treatment, both of which reduced SUMOylation, we didn’t notice any other significant change in Golgin45 SUMOylation, suggesting that Golgin45 SUMOylation by SUMO1 may also be modestly influenced by autophagy, which requires further study.

To investigate whether Golgin45 SUMOylation is restricted to SUMO1 only, we transfected HeLa cells with either Ubc9 WT or DN along with mCherry-Golgin45 and myc-SUMO1 or myc-SUMO3, followed by western analysis. As expected, Ubc9 DN failed to induce higher molecular weight SUMOylated Golgin45 bands (Fig. 1b; compare lane 1 and 2). Interestingly, we detected evidence of Golgin45 SUMOylation by SUMO3 as well as SUMO1, although SUMO3 modification was much weaker than SUMO1 under steady state condition (Fig. 1b; compare lane 2 and 4).

We then decided to search for potential SUMOylation sites on Golgin45 protein. We first use a SUMOylation prediction program, GPS-SUMO^19^, to find consensus SUMOylation sequence in Golgin45. K106 was found as a consensus SUMOylation sequence and K9 was found as an inverted SUMOylation consensus motif in Golgin45. To narrow down the search for potential SUMOylation sites on Golgin45 protein, we first checked the SUMOylation level of the truncated Golgin45. Since C-terminal deletion may cause disruption of Golgin45 targeting to the Golgi^20^, we initially compared N-terminal deletion mutant of Golgin45 (121–400) with the full length mCherry-Golgin45. HeLa cells were transfected with these two Golgin45 constructs along with FLAG-Ubc9 WT (or DN) and myc-SUMO1. Strikingly, Golgin45 (121–400) failed to show the typical SUMOylated Golgin45 upper bands (Fig. 1c; compare lane 1 and 2), suggesting that the majority of SUMOylation sites likely occur in the N-terminal one third of Golgin45 protein. These results allowed us to narrow down the potential lysine residues to mutate in a mutagenesis study to identify the SUMOylation sites on Golgin45.

Next, each lysine (K) residue in the N-terminal region of Golgin45 were mutated to arginine (R) and we performed a mutagenesis study using mCherry-Golgin45, Flag-Ubc9 and myc-SUMO1 to search for SUMOylation sites on Golgin45. The results showed that mutation of lysine residues at positions 4, 9, 67, 72, 77, 100, 106, 127 largely eliminated the multiple SUMOylated bands (Fig. 1d, e; compare lane 2 and 9). This eight-lysine mutant (8-KR) also failed to show the multiple SUMOylated bands, when the cells were transfected with either myc-SUMO1 or myc-SUMO3, indicating that SUMO1 and SUMO3 likely share similar SUMOylation sites for Golgin45 to a significant extent (Fig. 1f–h).

Next, to test whether Golgin45 SUMOylation is monomeric or multimeric in its nature, HeLa cells were transfected with either myc-SUMO1 K7/16/17R or myc-SUMO3-K11R that block multimer formation of these SUMO isoforms^1,9,21^ along with Flag-Ubc9 and mCherry-Golgin45 overnight, followed by western analysis. The results showed that expression of myc-SUMO1-K7/16/17R had no effect on Golgin45 SUMOylation, compared to the control myc-SUMO1, whereas my-SUMO3-K11R greatly reduced SUMO3 conjugation to Golgin45 (Fig. 1i, j). These results suggested that SUMO1 modification on Golgin45 is likely to be monomeric, while SUMO3 modification is likely to be multimeric and/or branched in its nature.

### SUMOylation enhances Golgin45 protein stability by inhibiting TNKS1-dependent PARylation of Golgin45

We have recently shown that Golgin45 protein stability is under strict regulation by TNKS1-dependent PARylation and subsequent proteasomal degradation^17^. In order to study whether there exists an interplay between SUMOylation and PARylation of Golgin45, HeLa cells were transiently transfected with mCherry-Golgin45 and SUMO1 along with Ubc9 WT or DN, followed by treatment with either DMSO or XAV939 (a specific inhibitor of TNKS1/2)^17^.

**Fig. 2:**
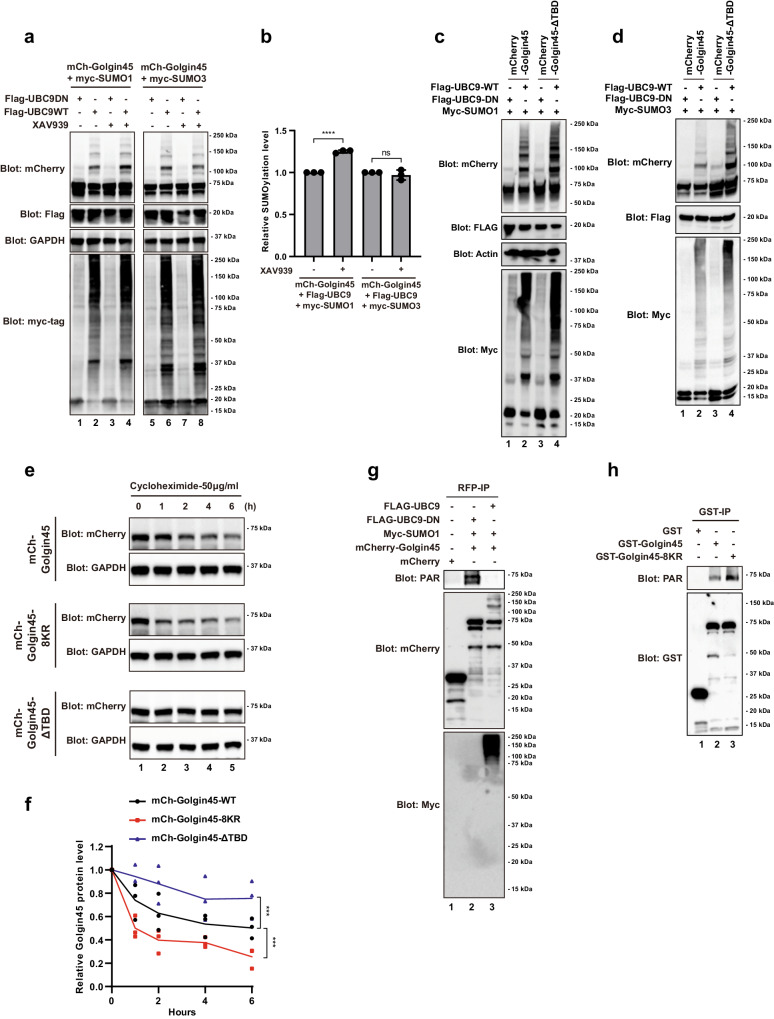
SUMOylation enhances Golgin45 protein stability by inhibiting TNKS1-dependent PARylation of Golgin45. **a** XAV939 treatment increases SUMO1-modified SUMOylation on Golgin45. HeLa cells were transiently transfected with mCherry-Golgin45 and SUMO1 or SUMO3 along with Ubc9 WT or DN, followed by either DMSO or XAV939 (a specific inhibitor of TNKS1/2). The cells were lysed and analyzed by western blotting using antibodies against the indicated proteins. **b** Relative levels of SUMO1 or SUMO3 conjugated mCherry-Golgin45 are presented as mean ± SD. Statistical analysis of relative SUMOylation was performed by one-way ANOVA with Tukey’s multiple comparisons. *n* = 3 independent experiments. ns: not significant. ****P < 0.0001. **c**, **d** Deletion of TNKS1 binding domain (ΔTBD) increases both SUMO1 and SUMO3 modified SUMOylation of Golgin45. After transfection with the indicate plasmids, HeLa cells were lysed and analyzed by western blotting using antibodies against the indicated proteins. **e**, **f** HeLa-Golgin45-KO cells stably expressing mCherry-Golgin45, mCherry-Golgin45-8KR mutant or mCherry-Golgin45-ΔTBD mutant were treated with cycloheximide (50 μg/ml) for the indicated times. Ectopically expressed WT and mutant mCherry-Golgin45 protein levels were analyzed by immunoblotting. Quantification of exogenous protein levels relative to GAPDH expression is shown in (**f**). Relative protein levels of mCherry-Golgin45 are presented as mean ± SD. Statistical analysis of relative protein level was performed by two-way ANOVA with Dunnett’s post-hoc test for multiple comparisons. *n* = 3 indepe*n*dent experiments. ***P < 0.001. **g** SUMOylation of mCherry-Golgin45 inhibits its PARylation. Hela cells transfected with indicate plasmids were lysed and immunoprecipitated using anti-RFP beads, and blotted with either anti-mCherry, anti-myc or anti-PAR. **h** Golgin45 PARylation is increased for Golgin45 8-KR mutant. HeLa cells expressing GST, GST-Golgin45 or GST-Golgin45-8KR were lysed and pulled down using GST beads, and analyzed by western blotting using anti-GST or anti-PAR antibodies. Representative blots are shown and experiments were repeated three times.

The results showed that XAV939 treatment greatly increased SUMO1-modified SUMOylation on Golgin45, but showed no effect on SUMO3-modified SUMOylation on Golgin45 (Fig. 2a, b; compare SUMOylated upper bands in lane 2 and 4 for SUMO1 and lane 6 and 8 for SUMO3). Deletion of TNKS1 binding domain (ΔTBD) showed a similar increase in Golgin45 SUMOylation as XAV939 treatment (Fig. 2c; lane 2 and 4), although deletion of TNKS1 binding domain also increased overall Golgin45 protein stability. Similar increase in SUMO3 conjugation was also observed in Golgin45 ΔTBD mutant (Fig. 2d). Taken together, these results suggested that inhibition of TNKS1 activity likely enhances Golgin45 SUMOylation.

As increased SUMOylation of Golgin45 in XAV939-treated cells and Golgin45 ΔTBD mutant appeared to coincide with overall enhancement of Golgin45 protein stability, we attempted to measure the changes in its protein half-life that can be more directly attributed to SUMOylation only. To this end, Golgin45-KO HeLa cells were stably expressed with mCherry-Golgin45 WT, Golgin45 ΔTBD mutant or Golgin45 (8-KR) mutant, followed by the Cycloheximide (CHX) protocol as described in the methods. The results indicated that Golgin45 ΔTBD mutant greatly enhanced protein stability of Golgin45. Golgin45 (8-KR) mutant, a SUMO-deficient mutant, showed decreased protein half-life, as shown in Fig. 2e, f, compared to the WT control, demonstrating that SUMOylation indeed enhances Golgin45 protein stability on its own.

Furthermore, we found using anti-PAR western blots that mCherry-Golgin45 PARylation is inhibited by increased SUMOylation via co-transfection of Flag-Ubc9 and myc-SUMO1 (Fig. 2g; compare lane 2 and 3). We also observed that PARylation is greatly increased for Golgin45 8-KR mutant, compared to the WT control (Fig. 2h; compare lane 2 and 3). Taken together, these results indicated that SUMOylation and PARylation of Golgin45 are likely to be in a dynamic interplay under steady state condition, providing an elaborate regulatory mechanism for Golgin45 protein stability.

### Inhibition of Golgin45-Importinβ2 binding abrogates Golgin45 SUMOylation

It is well known that SUMOylation plays crucial roles in the nucleus, such as PML-NB formation under cellular stress^1,15^. Golgin45 was originally identified as a nuclear co-factor, JEM-1, that is up-regulated during retinoid-induced maturation of NB4 promyelocytic leukemia, prior to its characterization as a Golgi structural protein, Golgin45^20,22^. We reasoned that SUMOylation of Golgin45 might be relevant to its localization and/or function in the nucleus, as opposed to TNKS1-mediated PARylation of Golgin45 providing its regulatory mechanism at the Golgi^17^.

**Fig. 3:**
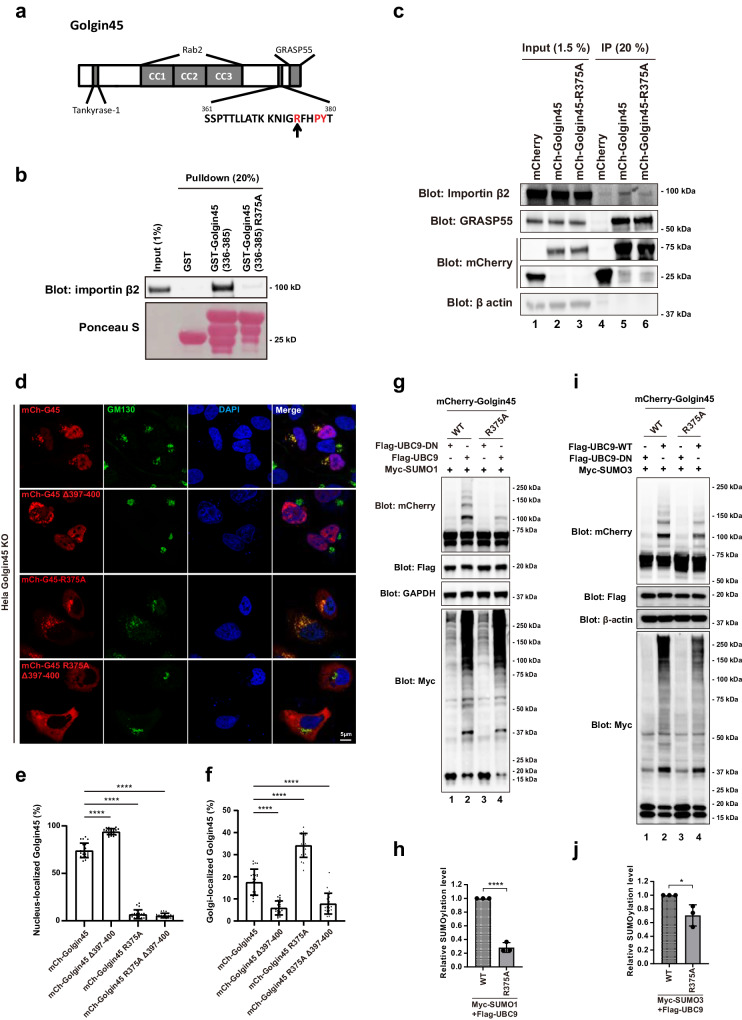
Importin-β2-mediated nuclear import of Golgin45 plays an important role in Golgin45 SUMOylation. **a** Domain structure of Golgin45. Tankyrase-1, Rab2-GTP, and GRASP55 bind to the N-terminus, coiled coil domain, and the C-terminus of Golgin45, respectively. The putative NLS on Golgin45 for importin β2 is noted with an arrow indicating a key residue R375. **b** R375A mutation greatly reduced the binding between Importin β2 and Golgin45. Purified His-tagged importin β2 was incubated with GST, GST-Golgin45 336–385 and GST-Golgin45 336–385 R375A and the bound fractions were analyzed by western blots using anti-importin β2 antibody. **c** Importin β2 was co-immunoprecipitated by Golgin45, but not R375A mutant. HeLa cells were tranfected with mCherry, mCherry-Golgin45 (mCh-G45), or mCherry-Golgin45 R375A (mCh-G45 R375A) for 18 h. Cells were lysed, incubated with anti-RFP beads, and analyzed with indicated antibodies. **d** mCherry-Golgin45, but not the R375A mutant, localized to the nucleus. HeLa cells overexpressing mCherry-Golgin45 or mCherry-Golgin45 Δ397–400, mCherry-Golgin45 R375A or mCherry-Golgin45 R375A Δ397–400 mutant were fixed and stained with anti-GM130 antibody. Images were acquired using Zeiss LSM880 confocal microscope. Statistical analysis was performed by one-way ANOVA to quantify the fluorescent intensity ratio of nucleus-(**e**) or Golgi-(**f**) localized Golgin45 over the total mCherry-Golgin45. **g**–**j** R375A mutation decreases SUMOylation of Golgin45. HeLa cells expressing mCherry-Golgin45, or mCherry-Golgin45-R375 mutant with SUMO1 (**g**) or SUMO3 (**i**) and UBC9 were lysed and SUMOylation of Golgin45 were analyzed by western blotting. Relative SUMOlation level of Golgin45 was analyzed by one-way ANOVA with Tukey’s multiple comparisons (**h**, **j**). *n* = 3 independent experiments. *P < 0.05.****P < 0.0001.

Thus, we decided to look for the nuclear localization signal (NLS) of Golgin45, in order to better understand its SUMOylation mechanism and nuclear import. A careful search for NLS led to identification of a non-classical PY NLS (^375^**RxxPY**^379^) in the C-terminal region of Golgin45^23^, as illustrated in Fig. 3a. We then expressed and purified a Golgin45 fragment (amino acids 336–385) fused to GST from bacteria and performed GST-pulldown assays with GST-Golgin45 (336–385) WT or the R375A mutant and purified Importin β2. The results showed that R375A mutation completely abrogates Golgin45 binding to Importin β2 (Fig. 3b; compare lane 3 and 4). Immunoprecipitation experiments confirmed this finding, although there was still some residual binding between R375A mutant and Importin β2 (Fig. 3c; compare lane 5 and 6).

The C-terminus of Golgin45 is required for Golgi targeting. Deletion of the C-terminus of Golgin45 (Golgin45 Δ397–400) lead to its translocalization to the nucleus. To confirm the important role of PY-NLS in controlling nuclear import of Golgin45, HeLa cells were transfected with either mCherry-Golgin45 or mCherry-Golgin45 Δ397–400, mCherry-Golgin45 R375A or mCherry-Golgin45-R375A Δ397–400 mutant overnight, followed by indirect staining with anti-GM130 (a Golgi marker) antibody and DAPI (nuclear staining). The results showed that mCherry-Golgin45 WT showed intense fluorescence both at the Golgi and in the nucleus, while mCherry-Golgin45 Δ397–400 localized to the nucleus only. Golgin45-R375A mutant showed strong fluorescence at the Golgi, while Golgin45-R375A Δ397–400 mutant showed diffused cytosol localization. Both Golgin45-R375A and Golgin45-R375A Δ397–400 mutant failed to localize to the nucleus, as expected (Fig. 3d–f).

Strikingly, R375A mutation almost eliminated SUMO1 conjugation to Golgin45, as shown in Fig. 3g, h, suggesting that Importin β2-mediated nuclear targeting is required for efficient Golgin45 SUMOylation by SUMO1. Similarly, SUMO3 conjugation to Golgin45 was also reduced for R375A mutant (Fig. 3I, j). These results demonstrate that Importin-β2-mediated nuclear import of Golgin45 plays an important role in Golgin45 SUMOylation.

### Heat shock stress greatly increases Golgin45 conjugation to SUMO2/3 in the nucleus

**Fig. 4:**
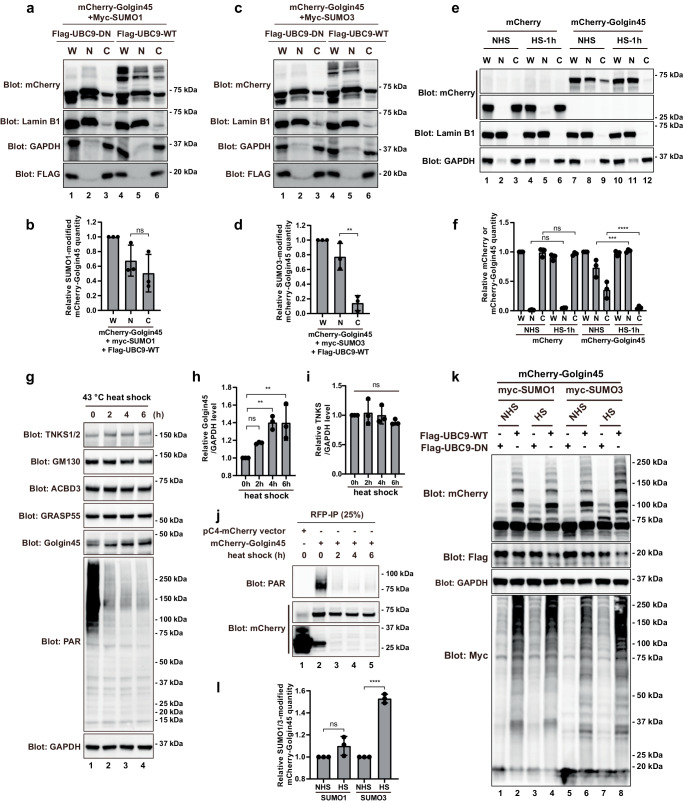
Heat shock stress increases protein level and SUMOylation of Golgin45. **a**–**d** HeLa cells expressing myc-SUMO1 (**a**) or SUMO3 (**b**) Flag-UBC9-WT or DN mutation and mCherry-Golgin45 were subjected to subcellular fractionation. The levels of SUMOylated Golgin45 in nuclear and cytoplasmic fractions were determined by immunoblotting with anti-mCherry antibody. The relative purity of the nuclear and cytoplasmic fractions was confirmed by sequential probing for the nuclear marker lamin B and the cytoplasmic marker GAPDH. W, whole cell lysate; C, cytoplasm; N, nucleus. Relative levels of SUMO1-(**b**), or SUMO3-(**d**) modified mCherry-Golgin45 were analyzed by one-way ANOVA with Tukey’s multiple comparisons. *n* = 3 independent experiments. ns: not significant. **P < 0.01. **e**, **f** Heat shock increases nuclear localization of Golgin45. HeLa cells expressing mCherry or mCherry-Golgin45 were treated with heat shock (HS) or no heat shock (NHS) for 1 h, and then subjected to subcellular fractionation. The levels of Golgin45 in nuclear and cytoplasmic fractions were determined by immunoblotting with anti-mCherry antibody. Percentage of mCherry and mCherry-Golgin45 in nucleus and cytoplasm was analyzed by two-way ANOVA with Dunnett’s post-hoc test for multiple comparisons. *n* = 3 indepe*n*dent experiments (**f**). ns: not significant. ***P < 0.001. ****P < 0.0001. **g**–**i** Heat shock increases endogenous protein level of Golgin45. HeLa cells were treated with heat shock for the indicated time points and harvested for western blots using indicated antibodies (**g**). Relative protein levels of endogenous Golgin45 (**h**) and TNKS (**i**) were analyzed by one-way ANOVA with Tukey’s post-hoc test for multiple comparisons. *n* = 3 independent experiments. ns: *n*ot significant. **P < 0.01. **j** Heat shock inhibits PARylation of Golgin45. HeLa cells expressing mCherry or mCherry-Golgin45 were treated with heat shock for indicated time points. Then cells were lysed and immunoprecipitated using anti-RFP beads and blotted with either anti-mCherry or anti-PAR antibodies. **k**, **l** Heat shock increases SUMO3 modified SUMOylation of Golgin45. HeLa cells expressing myc-SUMO1 or SUMO3, Flag-UBC9-WT or DN mutation and mCherry-Golgin45 were treated with or without heat shock for 1 h. Cells were harvested for western blots using indicated antibodies (**k**). Relative levels of SUMOylated mCherry-Golgin45 were analyzed by two-way ANOVA with Dunnett’s post-hoc test for multiple comparisons. *n* = 3 independent experiments (i). ns: not sig*n*ificant. ****P < 0.0001.

In order to test the possibility that SUMOylation itself may also positively reinforce Golgin45 import and retention in the nucleus, HeLa cells were transfected with mCherry-Golgin45, myc-SUMO1 (or SUMO3) and FLAG-Ubc9 WT or DN overnight. Cells were then analyzed via subcellular fractionation assays followed by immunoblotting. The results showed that Golgin45 was both in the nuclear and the cytoplasmic fractions for cells transfected with SUMO1 (Fig. 4a, b).

Strikingly, SUMOylated Golgin45 was almost exclusively found in the nuclear fraction in cells transfected with SUMO3 (Fig. 4c, d; compare upper bands in lane 5 and 6 in Fig. 4C), suggesting that SUMO3 modification may further enhance nuclear retention of Golgin45, compared to SUMO1 modification. This finding is consistent with previous reports that SUMO1 and SUMO3 modification play functionally distinct roles for SUMOylated proteins^24,25^.

As SUMOylation has been associated with stress response pathways^26^, we then posited that cellular stress may play a role in Golgin45 targeting to the nucleus. In particular, we asked whether heat shock stress may be linked to Golgin45 import into the nucleus, especially since Golgin45 protein level influences protein secretion and maturation at the Golgi, which had been known to be profoundly influenced by temperature^17,27,28^.

To study whether heat shock stress may influence nuclear import of Golgin45, HeLa cells were transfected with either control mCherry or mCherry-Golgin45 overnight, followed by heat shock at 43 °C (or control 37 °C) for 1 h. The cells were subjected to the same subcellular fractionation assays followed by immunoblotting.

The results showed that cytosolic fraction of mCherry-Golgin45 was greatly reduced after 1 h of heat shock treatment (Fig. 4e, f; compare lane 9 and 12 in Fig. 4e), whereas there was no obvious change in control mCherry protein. Consistent with this reduction in the cytosolic fraction, there was a corresponding increase in nuclear fraction of mCherry-Golgin45 (Fig. 4e; compare lane 8 and 11), indicating increased nuclear import of Golgin45 under heat shock stress. Overall, these results suggested that nuclear localization of Golgin45 likely increases under heat shock stress.

Next, we checked whether heat shock stress influences the stability of Golgin45 protein by western blots against endogenous Golgin45. To this end, HeLa cells were subjected to heat shock stress for 0, 2, 4 and 6 h, followed by cell lysis in SDS sample buffer and western analysis using indicated antibodies (Fig. 4g–i). The results showed that endogenous Golgin45 protein level increased upon heat shock stress (Fig. 4g–i). As controls, we also blotted for endogenous GM130 and known binding partners of Golgin45, including ACBD3, GRASP55 and TNKS1 as well as GAPDH (loading control), all of which showed no significant changes after heat shock treatment (Fig. 4g, i). In contrast, the total poly-ADP-ribose (PAR) level was dramatically reduced after heat shock. These results suggested that heat shock stress may influence Golgin45 import into the nucleus and protein stability of Golgin45, by altering Golgin45 SUMOylation and/or PARylation.

In support of this observation, we found that heat shock stress greatly reduces PARylation of mCherry-Golgin45, as shown in Fig. 4j. While we did not detect any PARylation of control mCherry alone, mCherry-Golgin45-transfected sample showed very broad and intense bands with apparent molecular weight around ~80–90 kDa, which are slightly above expected mCherry-Golgin45 band typically at ~75 kDa. Strikingly, these intense bands disappeared completely after heat shock treatment for 2, 4 and 6 h, suggesting that TNKS1 modulation of Golgin45 stability is highly temperature sensitive (Fig. 4j; compare lanes 2 and 3–5).

In order to investigate how heat shock stress may influence Golgin45 SUMOylation, HeLa cells were transfected with mCherry-Golgin45, myc-SUMO1 (or SUMO3) and FLAG-tagged Ubc9 WT or DN overnight, followed by 43 °C heat shock for 1 h. Cells were then lysed in RIPA buffer and subjected to western analysis. Unexpectedly, heat shock stress greatly increased SUMO3 modification (but not SUMO1 modification) on Golgin45 (Fig. 4k, l; compare lanes 2 and 4 in Fig. 4k), demonstrating that SUMO3 conjugation to Golgin45 may play more important roles in heat shock stress response, whereas SUMO1 modification may be relevant to housekeeping function of Golgin45 in the nucleus.

### PML directly binds Golgin45 and facilitates SUMO2/3 conjugation, facilitating Golgin45 inclusion into PML-NBs during heat shock response

Since Golgin45 was originally identified as a nuclear protein that is up-regulated during retinoid-induced maturation of NB4 promyelocytic leukemia (PML) and our results indicate that it appears to function as a stress response protein^10,15,22^, we wondered whether SUMOylated Golgin45 may directly interact with PML and participate in the PML nuclear body formation and its function in genome maintenance during cellular stress.

**Fig. 5:**
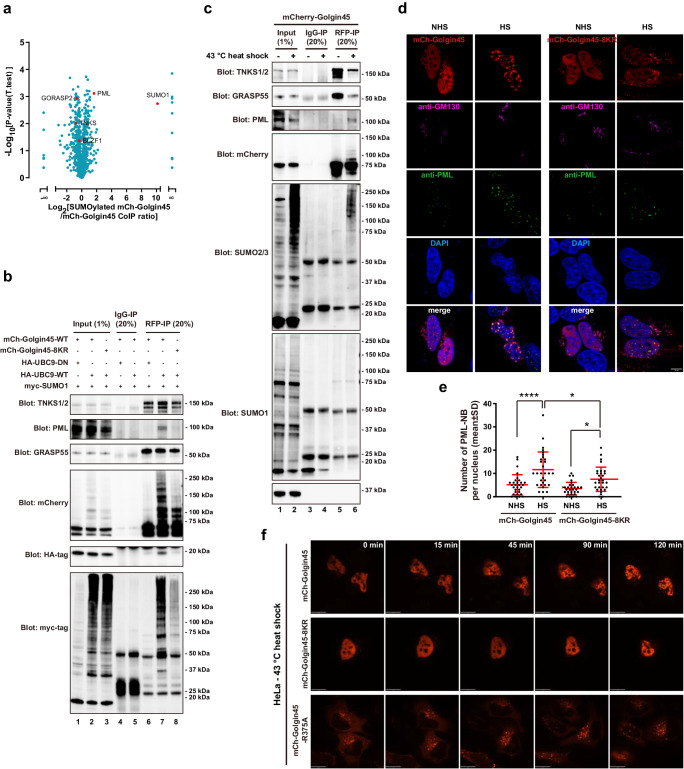
Heat shock stress increases SUMOylation of Golgin45 and promotes its inclusion into PML nuclear bodies. **a** Volcano plot representing results of the label-free IP-MS of SUMOylated mCherry-Golgin45. The logarithmic ratio of protein intensities in the SUMOylated mCherry-Golgin45/mCherry-Golgin45-CoIP were plotted against negative logarithmic *p* values of the t test performed from the triplicates. Indicated are known or candidate binding partners (red). **b** PML forms a complex with SUMOylated Golgin45. The protein extracts from HeLa cells expressing myc-SUMO1, HA-UBC9-DN/WT and mCherry-Golgin45-WT/8-KR were immunoprecipitated with anti-RFP beads. These lysates and the immunoprecipitates were analyzed by western blotting using anti-mCherry antibody and antibodies against the indicated proteins. **c** HeLa cells expressing mCherry-Golgin45 were treated with or without heat shock. The protein extracts were immunoprecipitated with anti-IgG or anti-RFP beads. These lysates and the immunoprecipitates were analyzed by western blotting using antibodies against the indicated proteins. **d** HeLa cells expressing mCherry-Golgin45 were treated with heat shock (HS) or no heat shock (NHS) for 2 h. Cells were then fixed and stained with anti-PML and anti-GM130 antibodies and DAPI. Scale bar, 10 μm. **e** Number of PML-NB per nucleus in (**d**) was quantified. Statistical analysis was performed using one-way ANOVA with a Tukey’s post-hoc test (*p < 0.05; ****p < 0.0001). N = 30 cells. **f** HeLa cells expressing mCherry-Golgin45 WT, R375A or 8-KR mutant were monitored by live cell imaging acquired every 5 min for 2 h at 43 °C. Imaging sequences at the indicated time points are presented here. Scale bar, 14 μm.

As a preliminary study to survey interacting proteins of SUMOylated Golgin45, HeLa cells were co-transfected with mCherry-Golgin45 and with/without SUMO1 and Ubc9, followed by cell lysis in RIPA buffer and immunoprecipitation using anti-RFP antibody. We then analyzed the immunoprecipitates by proteomic analysis. The results showed that PML protein showed at least threefold higher abundance in SUMOylated Golgin45 immunoprecipitates, compared to the control mCherry-Golgin45 (Fig. 5a, Supplementary Data 1).

To confirm this finding, HeLa cells were transfected with mCherry-Golgin45 or 8-KR mutant with SUMO1 and UBC9-DN/WT, followed by lysis in RIPA buffer and immunoprecipitation using anti-RFP beads. Western analysis of these immunoprecipitates confirmed that endogenous PML clearly prefers to interact with SUMOylated Golgin45, whereas the interactions between Golgin45 and GRASP55 or TNKS are not affected by the SUMOylation level of Golgin45 (Fig. 5b; compare lane 6, 7 and 8).

In support of this finding, we found that mCherry-Golgin45 pulls down increased amount of endogenous PML and reduced amount of TNKS1 and GRASP55 upon heat shock stress (Fig. 5c). We also observed that heat shock treatment induces greatly increased SUMO2/3 conjugation to various proteins overall as reported, whereas there was no significant change in SUMO1 modification (Fig. 5c, lane 1 and 2). Importantly, heat shock treatment induced SUMO2/3 conjugation to mCherry-Golgin45, which were reflected in increased higher MW upper bands in anti-mCherry and anti-SUMO2/3, but not in anti-SUMO1 western blots (Fig. 5c, compare lane 5 and 6). Taken together, these results further support the notion that heat shock stress greatly enhances Golgin45 SUMOylation by SUMO2/3 and its binding to PML protein.

In order to check whether SUMOylated Golgin45 is a part of PML-NBs in the nucleus under heat shock stress, HeLa cells were transfected with mCherry-Golgin45 overnight, followed by 43 °C heat shock for 2 h. Cells were then fixed and stained with anti-PML and GM130 antibodies and DAPI. Upon examining under confocal microscope, we found that mCherry-Golgin45 indeed co-localizes with the PML-NBs under heat shock stress, as shown Fig. 5d, e. Unexpectedly, however, we found that over-expression of SUMOylation-deficient Golgin45 8KR mutant reduced the number of PML-NBs after heat shock treatment (Fig. 5d, e), suggesting that the over-expressed 8KR mutant may function as a dominant negative for PML-NB formation.

These results were further validated using live cell imaging. To this end, cells were transfected with mCherry-Golgin45 or Golgin45-8KR mutant or Golgin45-R375A mutant overnight, followed by live cell imaging using a confocal microscope for 2 h under heat shock stress. These results were consistent with the earlier observation using fixed samples in that there was gradual and condensation of mCherry-Golgin45 WT into distinct puncta, while the 8KR or R375A mutant didn’t show this condensation of red fluorescence during the 2 h live cell imaging under heat shock condition (Fig. 5f).

### Endogenous Golgin45 is SUMOylated and translocates to the nucleus upon heat shock

**Fig. 6:**
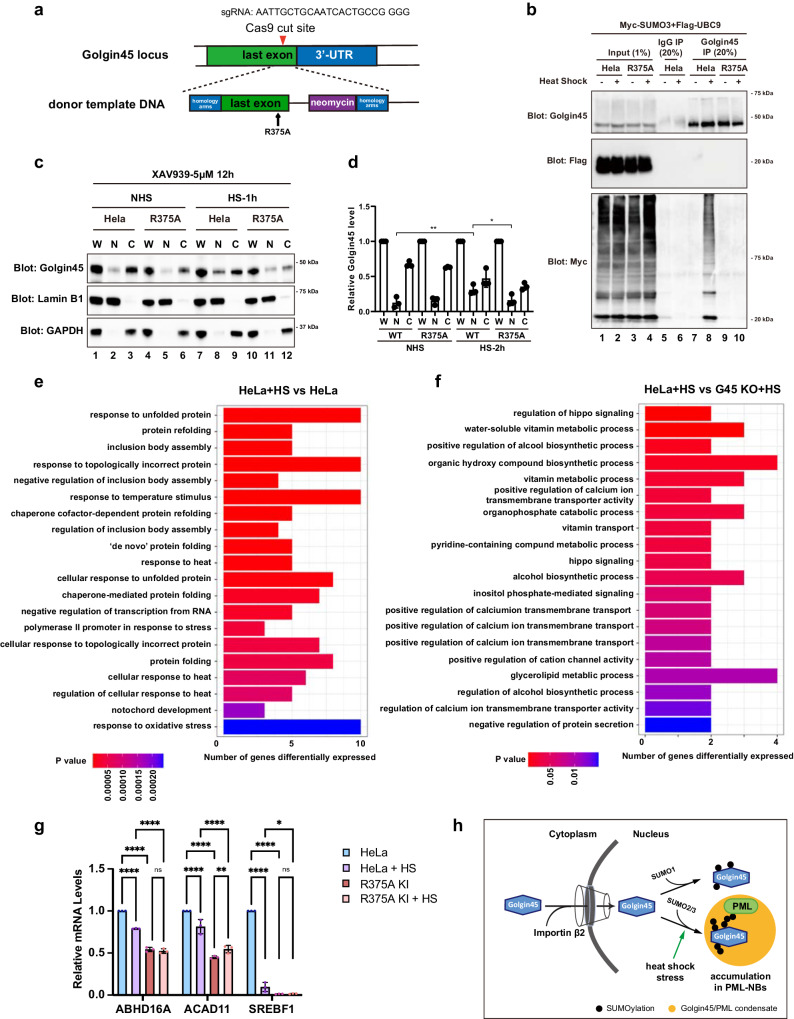
Endogenous Golgin45 enters the nucleus and regulates gene transcription in response to heat stress. **a** Schematic illustration of CRISPR-Cas9-mediated mutation of R375A in endogenous Golgin45. **b** HeLa wildtype and R375 knockin cells expressing myc-SUMO3 and Flag-UBC9 were subjected to immunoprecipitation using anti-Golgin45 antibody. **c** HeLa wildtype and R375A knockin cells were subjected to subcellular fractionation. Cells were treated with 5 μM XAV939 for 12 h, prior to heat shock (HS) or no heat shock (NHS) treatment for 2 h. The cytoplasmic and nuclear fractions of cell lysis were determined by western blotting using specific antibodies. W, whole cell lysate; C, cytoplasm; N, nucleus. **d** Relative protein level of Golgin45-WT or R375A in nucleus and cytoplasm compared to their own WCL was analyzed by two-way ANOVA with Dunnett’s post-hoc test for multiple comparisons. *n* = 3 independent experiments. **P < 0.01. *P < 0.05. **e**, **f** Gene expression profiling of HeLa wildtype and Golgin45 KO cells before and after heat shock. Significantly upregulated genes in HeLa + HS vs. HeLa + NHS cells and HeLa + HS vs. HeLa Golgin45 KO + HS cells were subjected to GO enrichment analysis. **g** The transcription of *ABHD16A*, *ACAD11*, and *SREBF1* were reduced in R375KI cells. Indicated genes were tested for mRNA expression levels in HeLa wildtype, R375A KI cells with or without heat stress treatment. Relative enrichment of genes was quantified and analyzed by one-way ANOVA with Tukey’s multiple comparisons. *n* = 3 independent experiments. ***P < 0.001. ****P < 0.0001. **h** Model for Golgin45 SUMOylation and its role in cellular stress response. Golgin45 gets imported into the nucleus via its interaction with Importin-β2. Golgin45 is conjugated to SUMO1 under steady state condition. Cellular stress (such as heat shock) promotes SUMO2/3 conjugation to Golgin45, leading to its inclusion into PML-NBs.

Our results showed so far that SUMOylation of overexpressed mCherry-Golgin45 is enhanced during heat stress. To rule out the possibility of an overexpression artifact and study the localization and function of SUMOylated endogenous Golgin45, we prepared a R375A mutation on endogenous Golgin45 in HeLa cells using CRISPR/Cas9 technology as a negative control for the following experiments (Fig. 6a). The monoclonal cells successfully edited were analyzed via genomic sequencing (Supplementary Fig. 2).

HeLa wildtype and R375A knock-in cells were transfected with FLAG-Ubc9 and myc-SUMO3 and immunoprecipitated with anti-Golgin45 antibody. In line with our results using overexpressed Golgin45, endogenous Golgin45 was indeed SUMOylated under heat stress, whereas the R375A mutant was not (Fig. 6b), although the typical SUMOylated upper bands were difficult to detect. In line with our findings, however, a recent proteomics paper, which investigated SUMO proteome, also reported that Golgin45 is a SUMO-modified protein^29^.

As our results showed that overexpressed Golgin45 entered nucleus upon heat shock, we also tested whether endogenous Golgin45 behaves the same way. We incubated HeLa wildtype and R375 knock-in cells at 43 °C for 2 h, prior to separation of cytosolic and nuclear fractions. At steady state, endogenous Golgin45 was mostly found in the cytosolic fraction. After heat stress, a substantial amount of endogenous Golgin45 re-localized to the nucleus, while R375A mutant stayed in the cytosol (Fig. 6c, d), indicating that endogenous Golgin45 gets transported to the nucleus via its NLS upon heat shock.

### SUMOylated Golgin45 induces transcription of a subset of genes involved in lipid biosynthesis

To investigate the functional role of SUMOylated Golgin45 in heat stress response, we first tested whether enhanced SUMOylation of Golgin45 affects the trafficking of newly synthesized protein. To this end, we examined secretion of a commonly-used protein cargo, horseradish peroxidase containing a signal sequence (ss-HRP), in HeLa cells overexpressing mCherry (negative control), mCherry-Golgin45, or mCherry-Golgin45-8KR with or without heat shock treatment. The results indicated that the secretion of ss-HRP after heat shock was greatly reduced to ~10% of non-heat shock control groups (Supplementary Fig. 3). However, overexpression of mCherry-Golgin45, or mCherry-Golgin45-8KR did not have significant changes in the secretion of ss-HRP.

Since Golgin45 was identified as a nuclear factor and its nuclear fraction increases after heat shock, we next examined whether SUMOylated Golgin45 is important for gene transcription in response to heat stress. HeLa wildtype and Golgin45 knockout cells with and without heat shock treatment were analyzed by mRNA sequencing. As expected for a control, GO analysis of genes up-regulated in HeLa wildtype cells after heat stress compared to untreated cells showed that the prominent biological processes were linked to protein folding and stress response (Fig. 6e, Supplementary Data 2). Strikingly, transcription of genes induced by heat shock in wildtype cells compared to Golgin45 knockout cells were mostly involved in lipid biosynthesis and metabolism (Fig. 6f, Supplementary Data 3).

We further verified these results by testing the transcription of three lipid metabolism-related genes, *ABHD16A* (abhydrolase domain-containing protein 16A), *ACAD11* (acyl-CoA dehydrogenase family member 11), and *SREBF1* (sterol regulatory element binding transcription factor 1), identified by mRNA sequencing analysis. Quantification of mRNA samples purified from HeLa wildtype, Golgin45-KO and R375A knock-in cells with or without heat shock treatment were performed by quantitative real time PCR. The results showed that heat shock stress reduced transcription of *ABHD16A* and *ACAD11* by ~20%, whereas the transcription of *SREBF1* in HeLa cells after heat stress was only ~10% of untreated cells. In R375A knock-in cells, transcription of *ABHD16A* and *ACAD11* decreased by ~50% over the wildtype cells, while *SREBF1* was barely transcribed at all. Heat shock did not further reduce the transcription level of all three genes tested in R375A knock-in cells (Fig. 6g).

Taken together, these results strongly suggest that (i) SUMOylated Golgin45 may influence lipid biosynthesis and metabolism during heat shock response by regulating relevant gene transcription; (ii) PML-NB interaction with SUMOylated Golgin45 may play a role in this process.

## Discussion

In this study, we showed that a Golgi structural protein, Golgin45, is modified with SUMO1 under steady state condition. Golgin45 harbors a NLS required for its import into the nucleus via its interaction with Importin-β2. Once in the nucleus, cellular stress (such as heat shock) likely further promotes SUMO2/3 conjugation to Golgin45, facilitating its inclusion into PML-NBs, as shown in the schematic illustration (Fig. 6h). As noted in the introduction, Golgin45 is also a PARylated protein via its interaction with TNKS1 at the Golgi^17^, and our results indicate that this post-translational modification seems negatively regulated by Golgin45 SUMOylation (Fig. 2g).

Several results support the hypothesis that Golgin45 may function as a stress response protein. First, heat shock stress increases stability of endogenous Golgin45 protein in a time-dependent manner (Fig. 4g). Second, SUMO3 conjugation to Golgin45 is highly stimulated under heat shock stress (Fig. 4k). Third, SUMO3 conjugation to Golgin45 greatly increases nuclear import and/or retention of Golgin45 in PML-NBs (Fig. 4c; Fig. 5c–e), suggesting PML-SUMO3-Golgin45 as a novel stress response pathway.

A growing body of evidence suggests that SUMO1 and SUMO2/3 may play distinct roles during stress response, in which SUMO2/3 provides a quick response to cellular stress. These include SUMOylation of NLRP3 during inflammatory response^25,30^, regulation of a non-canonical type I interferon response by SUMO2/3^31^ and differential effects of SUMO1 vs. SUMO3 on PKR activation^24^, etc.

It is also possible that SUMOylation of Golgin45 plays more prominent roles in specific cell-types, such as neuronal cells or cells adapted for heavy secretory activity, which requires further studies.

Our results showed that exogenous over-expression of Golgin45 (8-KR), a SUMO-deficient mutant, greatly reduced the formation of PML-NBs under heat shock stress (Fig. 5d). We don’t believe that these results indicate SUMOylated Golgin45 as an absolute requirement for PML-NB formation, but it does suggest that SUMOylated Golgin45 may contribute to the nucleation of PML-NBs during heat stress, which is supported by similar observations from earlier studies^32,33^.

Why would a Golgi structural protein like Golgin45 get SUMOylated and function as a component of PML-NBs upon heat stress, subsequently influencing lipid biogenesis and metabolism? It has been previously shown that certain PML-NBs regulate lipid metabolism via modulating assembly of nuclear lipid droplets (nLD) as a part of cellular stress response^34,35^. These nLD are known to be partly derived from the nucleoplasmic reticulum and its biogenesis also involves de novo lipid biosynthesis^34^. Thus, it makes sense that SUMOylated Golgin45 may interact with PML and PML-NBs and influences lipid metabolism upon heat stress.

On the other hand, protein SUMOylation has also been associated with cellular senescence via several senescence-related proteins, such as p53, TRF2 and SIRT1, etc, all of which are SUMOylated proteins^36–41^. In this respect, it is worth noting that TNKS1, a Golgin45 interacting protein, was originally identified as a PARP for TRF2 and its modulator in telomere maintenance^42–44^. Although it may be too speculative yet, these seeming connections raise a possibility that Golgin45 SUMOylation and PARylation may provide a Golgi-derived feedback mechanism for stress-induced cellular senescence^36^.

Finally, a previous screening study have shown that Histone Deacetylase-4 (HDAC4) may directly interact with both Golgin45 and TNKS1^45^. Since HDAC4 is known to function as a SUMO E3 ligase for SIRT1^46^, our results suggest that a more intricate network of post-translational modification-dependent regulation may exist along the secretory pathway for stress response than was previously thought. In summary, we propose, based on our results on PML-SUMO/Golgin45-TNKS1 interplay, that SUMOylated Golgin45 may play a role in cellular stress response by modulating lipid metabolism through interaction with PML-NBs.

## Methods

### Reagents and antibodies

All common reagents were purchased from Sigma-Aldrich, unless otherwise mentioned. XAV939 (S1180), MG132 (S2619), TAK981 (S8341), TAK243 (S8829), Doxorubicin (S1208) and Torin1 (S2827) were purchased from Selleck. Cycloheximide (100 mM) from Yeasen (40325ES03). The following antibodies were used: rabbit polyclonal anti-PML (21041-1-AP, proteintech, 1:1000 for WB), mouse monoclonal anti-PML (ab96051, Abcam, 1:500 for IF), rabbit polyclonal anti-mCherry (ab167453, Abcam, 1:2000 for WB), anti-GM130 (1:2000 for WB and 1:1000 for IF, ab52649, Abcam), mouse monoclonal anti-FLAG (F1804, Sigma-Aldrich, 1:2000 for WB), rabbit anti-Myc (2278S, Cell Signaling Technology, 1:2000 for WB), mouse anti-Actin (3700S, Cell Signaling Technology, 1:5000 for WB), anti-GST (RPN1236, Cytiva, 1:2000 for WB), rabbit polyclonal anti-Mono/poly-ADP Ribose (83732S, Cell Signaling Technology, 1:1000 for WB), rabbit polyclonal anti-Golgin45 (PA5-30714, Thermo, 1:1000 for WB), anti-ACBD3 (HPA015594, Sigma Aldrich, 1:2000 for WB), mouse monoclonal anti-GRASP55 (ab211532, Abcam, 1:1000 for WB), anti-GAPDH (KC-5G5, Kangchen Bio-tech, 1:5000 for WB), mouse monoclonal anti-Transportin 1 (ab10303, Abcam, 1:2000 for WB), rabbit polyclonal anti-Lamin B (12987-1-AP, Proteintech, 1:1000 for WB), mouse monoclonal anti-Tankyrase1/2 (sc-365897, Santa Cruz, 1:1000 for WB), mouse anti-HA-Tag (2367S, Cell Signaling Technology, 1:1000 for WB). Anti-Rabbit Alexa Fluor 488 (A21441, 1:500), Alexa Fluor 568 (A10042, 1:500), Alexa Fluor 647 (A21245, 1:500) and anti-Mouse Alexa Fluor 488 (A21200, 1:500), Alexa Fluor 568 (A10037, 1:500), Alexa Fluor 647 (A21236, 1:500) for Immunofluorescence were obtained from ThermoFisher.

### Cell culture and and transfection

HeLa (ATCC, CCL-2) cells were grown in DMEM supplemented with 10% FBS (Thermo) at 37 °C. HeLa cells were authenticated by STR profiling and were routinely tested for the mycoplasma contamination and were negative. Transfection of DNA constructs was performed using Lipofectamine 2000 (ThermoFisher), according to the manufacturer’s instructions. For DNA expression, cells were transfected 24 h before Co-IP experiments and 18 h for IF experiments.

### Co-immunoprecipitation (Co-IP) and Immunoblotting

For Co-IP experiments, total lysates were prepared using lysis buffer (25 mM HEPES, pH 7.4, 100 mM NaCl, 1% NP-40, 1× protease inhibitor cocktail (Roche)). Subsequently, the total lysates were passed through a syringe needle (15 times) and then incubated at 4 °C with end-over-end agitation for 1 h. The lysates were then cleared by centrifugation at 15,000 × *g* for 20 min. The supernatants were incubated with indicated antibodies and protein G agarose beads (L00209, GenScript) for 4 h at 4 °C with end-over-end agitation. The beads were washed two times with ice-cold lysis buffer and one time with PBS. Proteins were eluted by boiling in 2x SDS running buffer and subjected to SDS-PAGE for immunoblotting.

For immunoblotting, proteins were separated by SDS-PAGE (Genscript) and transferred onto nitrocellulose membranes (Amersham). Membranes were probed with specific primary antibodies and then with peroxidase-conjugated secondary antibodies (Jackson ImmunoResearch). The bands were visualized with chemiluminescence (Clarity Western ECL Substrate, Bio-Rad) and imaged by a ChemiDoc Touch imaging system (Bio-Rad) (Supplementary Fig. 4 for raw images of all blots).

### Immunofluorescence staining

Cells grown on glass coverslips in 24-well plates or glass bottom 24-well plates (P24-1.5H-N, Cellvis) were fixed for 10 min with 4% paraformaldehyde (PFA), permeabilized in permeabilization Buffer (0.3% Igepal CA-630, 0.05% Triton-X 100, 0.1% IgG-free BSA in PBS) for 5 min, and blocked in blocking buffer (0.05% Igepal CA-630, 0.05% Triton-X 100, 5% normal goat serum in PBS) for 60 min. Primary and secondary antibodies were applied in blocking buffer for 1 h. The nucleus was stained with Hoechst-33342 (sc-200908, Santa cruz Biotechnology). Cells were washed three times with wash buffer (0.05% Igepal CA-630, 0.05% Triton-X 100, 0.2% IgG-free BSA in PBS) and twice with PBS. Coverslips were mounted using ProLong Gold Antifade Reagent (ThermoFisher). Dip coverslip in diH_2_O before mounting to prevent salt contamination. Images were acquired with a Zeiss LSM880 confocal microscope using a 63x Apochromat oil-immersion objective.

### Live cell imaging

For live cell imaging of mCherry-Golgin45, R375A or 8-KR mutant, HeLa cells were seeded on a glass-bottom dish (35-mm diameter, Cellvis) coated with fibronectin (Millipore). After 18-h transfection, cells were imaged with a 100x objective on a Perkin Elmer UltraVIEW Spinning disk confocal microscope at 43 °C. Images were acquired every 5 min for 2 h.

### CRISPR/Cas9 gene editing

Gene-specific single-guide RNA (sgRNA) sequences were designed using the online software (http://crispr.mit.edu) resource from the Zhang Laboratory and were cloned into pSpCas9(BB)-2A-GFP (Addgene 48138) using the BbsI restriction enzyme sites. The target sequence was as follows: Golgin45 sgRNA, gttacttcatccccaatccgagg. The single-guide RNA DNA sequence was cloned into the pSpCas9(BB)-2A-GFP plasmid. HeLa cells were transfected with Golgin45 sgRNA-containing plasmid. After two days, isolation of clonal cell populations was performed by dilution into 96-well plate. Single clones were expanded and screened by immunoblotting, genomic sequencing, and functional assays.

### Subcellular protein fractionation

To produce subcellular fractionation, a cell subcellular fractionation kit (P0027, beyotime) was used. Briefly, cells were washed twice and lysed in buffer A plus 1 mM PMSF. The lysate was incubated at ice for 15 min. After incubation, buffer B was added to lysates and incubate at ice for 1 min. Then lysate was vortex 5 s and then centrifuged at 4 °C for 5 min at 16,000 × *g*. Supernatants were collected as cytoplasmic extracts, and pellets were collected as nuclear extracts. Equal amounts of cytoplasmic and nuclear extracts were subjected to western blot.

### Cycloheximide chase assay

The stability of Golgin45 was determined by cycloheximide chase assays. HeLa cells were seeded in six-well plates (Nest), then the cells were transiently transfected with mCherry-Golgin45, mCherry-Golgin45 8-KR mutant, mCherry-Golgin45 ΔTBD, mCherry-Golgin45-K350/370/371 R mutant for 18 h. Cells were treated with cycloheximide (50 μg/ml) for the indicated times and then collected and analyzed by Western blotting.

### GST pulldown assay

200 µg GST-fusion proteins were immobilized on glutathione Sepharose 4B (GE Healthcare) for each sample. The resins were washed 3 times with PBS + 0.5% Trition-X100 and then incubated with 0.1 μM Importin β2 proteins in pulldown buffer (25 mM Hepes, pH 7.37, 300 mM NaOAc, 25 mM Mg(OAc)2, 0.5% Triton X-100, 1 mM DTT and protease inhibitors, 500 μl total volumn for each reaction) at 4 °C for 2 h. The resins were washed 4 times with pulldown buffer. Samples were prepared by resuspension of the resins with 2x SDS loading buffer in 95 °C.

#### SS-HRP secretion assay

HeLa cells were co-transfected ss-HRP with mCherry (negative control), or mCherry-Golgin45, or mCherry-Golgin45-8KR with or without heat shock treatment, and then the conditioned media were harvested 18 h post-transfection. HRP activity was measured using 1-Step Ultra TMB-ELISA (ThermoFisher), according to the manufacturer’s instructions.

#### MS sample preparation, analysis and data analysis

HeLa cells expressing mCherry-Golgin45, Flag-UBC9-WT/DN and myc-SUMO1 were subjected to Co-IP experiment using RFP-beads (M165-8, MBL life science). Independent triplicates of mCherry-Golgin45 Co-IP experiments were prepared and analyzed by MS independently in a label free format. Samples were prepared by in-gel digestion as follows. Peptides were separated and analyzed on an Easy-nLC 1000 system coupled to a Q Exactive HF (both - Thermo Scientific). About 1 μg of peptides were separated in an home-made column (75 μm × 15 cm) packed with C18 AQ (5 μm, 300 Å, Michrom BioResources, Auburn, CA, USA) at a flow rate of 300 nL/min. Mobile phase A (0.1% formic acid in 2% ACN) and mobile phase B (0.1% formic acid in 98% ACN) were used to establish a 60 min gradient comprised of 2 min of 5% B, 40 min of 5–26% B, 5 min of 26–30% B, 1 min of 30–35% B, 2 min of 35–90% B and 10 min of 90% B. Peptides were then ionized by electrospray at 1.9 kV. A full MS spectrum (375–1400 m/z range) was acquired at a resolution of120,000 at m/z 200 and a maximum ion accumulation time of 20 ms. Dynamic exclusion was set to 30 s. Resolution for HCD MS/MS spectra was set to 30,000 at m/z 200. The AGC setting of MS and MS2 were set at 3E6 and 1E5, respectively. The 20 most intense ions above a 1.0E3 counts threshold were selected for fragmentation by HCD with a maximum ion accumulation time of 60 ms. Isolation width of 1.6 m/z units was used for MS2. Single and unassigned charged ions were excluded from MS/MS. For HCD, normalized collision energy was set to 25%.

The raw data were processed and searched with MaxQuant 1.5.4.1 with MS tolerance of 4.5 ppm, and MS/MS tolerance of 20 ppm. The UniProt human protein database (release 2016_07, 70630 sequences) and database for proteomics contaminants from MaxQuant were used for database searches. Reversed database searches were used to evaluate false discovery rate (FDR) of peptide and protein identifications. Two missed cleavage sites of trypsin were allowed. Carbamidomethylation (C) was set as a fixed modification, and oxidation (M), Acetyl (Protein N-term) and deamidation (NQ) were set as variable modifications. The FDR of both peptide identification and protein identification was set to be 1%^47^. The options of “Second peptides”, “Match between runs” and “Dependent peptides” were enabled. Label-free quantification was used to quantify the difference of protein abundances between different samples^48,49^.

### RNA-seq and analysis

Total RNA was prepared using Trizol reagent (Thermo) according to the manufacturer’s instructions. RNA integrity analysis, sample preparation, RNA-sequencing and analysis were performed by ApexBio technology. RNA quality was examined by 0.8% agarose gel electrophoresis and spectrophotometry. High-quality RNA with a 260/280 absorbance ratio of 1.8–2.2 was used for library construction and sequencing. Illumina library construction was performed according to the manufacturer’s instructions (Illumina, USA). Oligo-dT primers are used to transverse mRNA to obtain cDNA (APExBIO, Cat. No. K1159). Amplify cDNA for the synthesis of the second chain of cDNA. Purify cDNA products by AMPure XP system (Beckman Coulter, Beverly, USA). After library construction, library fragments were enriched by PCR amplification and selected according to a fragment size of 350–550 bp. The library was quality-assessed using an Agilent 2100 Bioanalyzer (Agilent, USA). The library was sequenced using the Illumina NovaSeq 6000 sequencing platform (Paired end150) to generate raw reads.

Raw paired-end fastq reads were filtered by TrimGaloreto discard the adapters and low quality bases via calling the Cutadapt tool. The clean reads obtained were then aligned to the mm10/hg19 human genome using HISAT2, followed by reference genome-guided transcriptome assembly and gene expression quantification using StringTie. Differentially expressed genes (DEGs) were identified by DEseq2 (for sample with replications) or edgeR (for sample with no replication) with a cut-off value of log2|fold-change| > 1 and p-adjust <0.05. The clusterProfiler was used to perform functional enrichment analysis for the annotated significant DEGs, the potential genes in identified modules based on gene ontology (GO) and KEGG pathway categories. Terms with p value < 0.05 were considered significant. Gene set enrichment analysis (GSEA) was performed by the function in package clusterProfiler with a gene list sorted by log2 fold-change.

### Quantitative real-time PCR analysis

HeLa-WT and R375A-KI cells were treated with or without heat shock for 2 h. Total RNA was prepared using Trizol reagent (Thermo). Afterwards, RNA samples were reverse transcribed using HiScript II Q RT SuperMix (R223-01, Vazyme). Real time-PCR was performed by a QuantStudio 7 real-time PCR system (ThermoFisher) using ChamQTM Universal SYBR qPCR Master Mix (Q711-02, Vazyme). GAPDH mRNA was used for normalization. The following specific primers were used: ABHD16A-forward: TGCCGTTTTCTCACTATGCTG; ABHD16A-reverse: CCGATGTGTTGCTTCCAAGATG; ACAD11-forward: TGCTACTGGCGAGTCCGAT; ACAD11-reverse: TGTGCTTTAGGAAGAAGTGAACC; SREBF1-forward: ACAGTGACTTCCCTGGCCTAT; SREBF1-reverse: GCATGGACGGGTACATCTTCAA; GAPDH-forward: ACCACAGTCCATGCCATCAC, GAPDH-reverse: TCCACCACCCTGTTGCTGTA.

### Statistics and reproducibility

Quantification of PML-NB number or quantification of the fluorescent intensity ratio of nucleus- or Golgi- localized Golgin45 were analyzed using Fiji software. All graphes were analyzed using the Prism GraphPad software (version 9.0) and statistical analysis was performed using one-way ANOVA with a Tukey’s post-hoc test or two-way ANOVA with Dunnett’s post-hoc test for multiple comparisons (n.s., not significant; *p < 0.05; **p < 0.01; ***p < 0.001; ****p < 0.0001). Data are presented as mean ± SD. All experiments were carried out in triplicates, unless specified otherwise.

### Reporting summary

Further information on research design is available in the Nature Portfolio Reporting Summary linked to this article.

### Supplementary information

Supplementary information

Description of Supplementary Materials

Supplementary data 1

Supplementary data 2

Supplementary data 3

Supplementary data 4

reporting summary

## Data Availability

The mass spectrometry proteomics data have been deposited to the ProteomeXchange Consortium via the PRIDE partner repository with the dataset identifier PXD051237. The RNA-Seq data presented in this study are available in the NCBI Sequence Read Archive (NCBI-SRA BioProject ID: PRJNA1097982). All materials and data supporting this study are available from the corresponding authors (yuexh@shanghaitech.edu; qianyi@shanghaitech.edu; intalee@gmail.com) upon reasonable request. Images of uncropped blots are provided in Supplementary Fig. 4. Source data for graphs are available in Supplementary Data 4.
