# Supplementary material for: SUMOylated Golgin45 associates with PML-NB to transcriptionally regulate lipid metabolism genes during heat shock stress
Source: Commun Biol. 2024 May 6;7:532. doi: 10.1038/s42003-024-06232-3 (PMC11074300; doi:10.1038/s42003-024-06232-3)

Supplementary figure 1

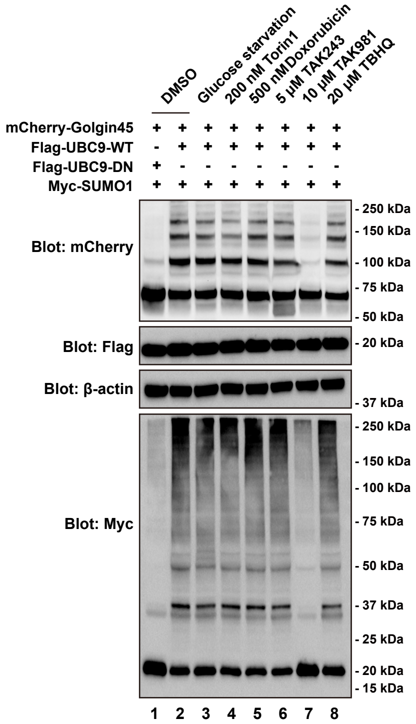

Supplementary Figure 1 HeLa cells were transiently transfected with the indicated plasmids overnight, then the cells were treated with DMSO, Glucose depletion, 500 nM Doxorubicin, 200 nM Torin1, 10 µM TAK243, 10 µM TAK981 or 20 µM TBHQ for 8 hours and then SUMO1-modified Golgin45 was analyzed by western blot.

Supplementary figure 2

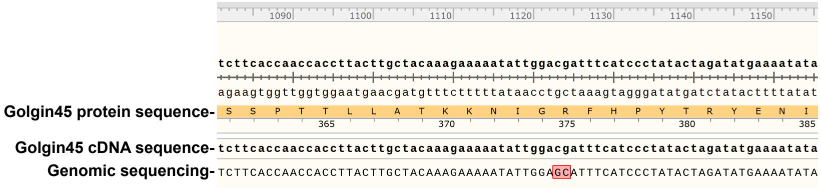

Supplementary Figure 2 Sequence alignment showing the mutated base pairs between 1123bp and 1124bp in Golgin45-R375A Knock-in HeLa cells.

Supplementary figure 3

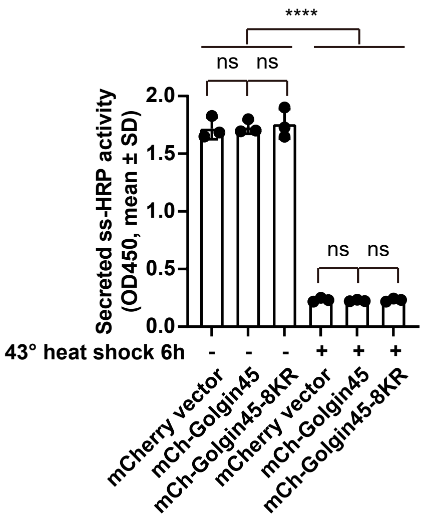

Supplementary Figure 3 HeLa cells co-expressing ss-HRP with mCherry (negative control), or mCherry-Golgin45, or mCherry-Golgin45-8KR with or without heat shock treatment, and then the secretion of ss-HRP after heat shock was detected.

Supplementary Figure 4
Unprocessed images of all blots

Unprocessed western blots in main figures. Some blots were cut into several pieces and incubated with different antibodies.

Unprocessed blots of Figure 1

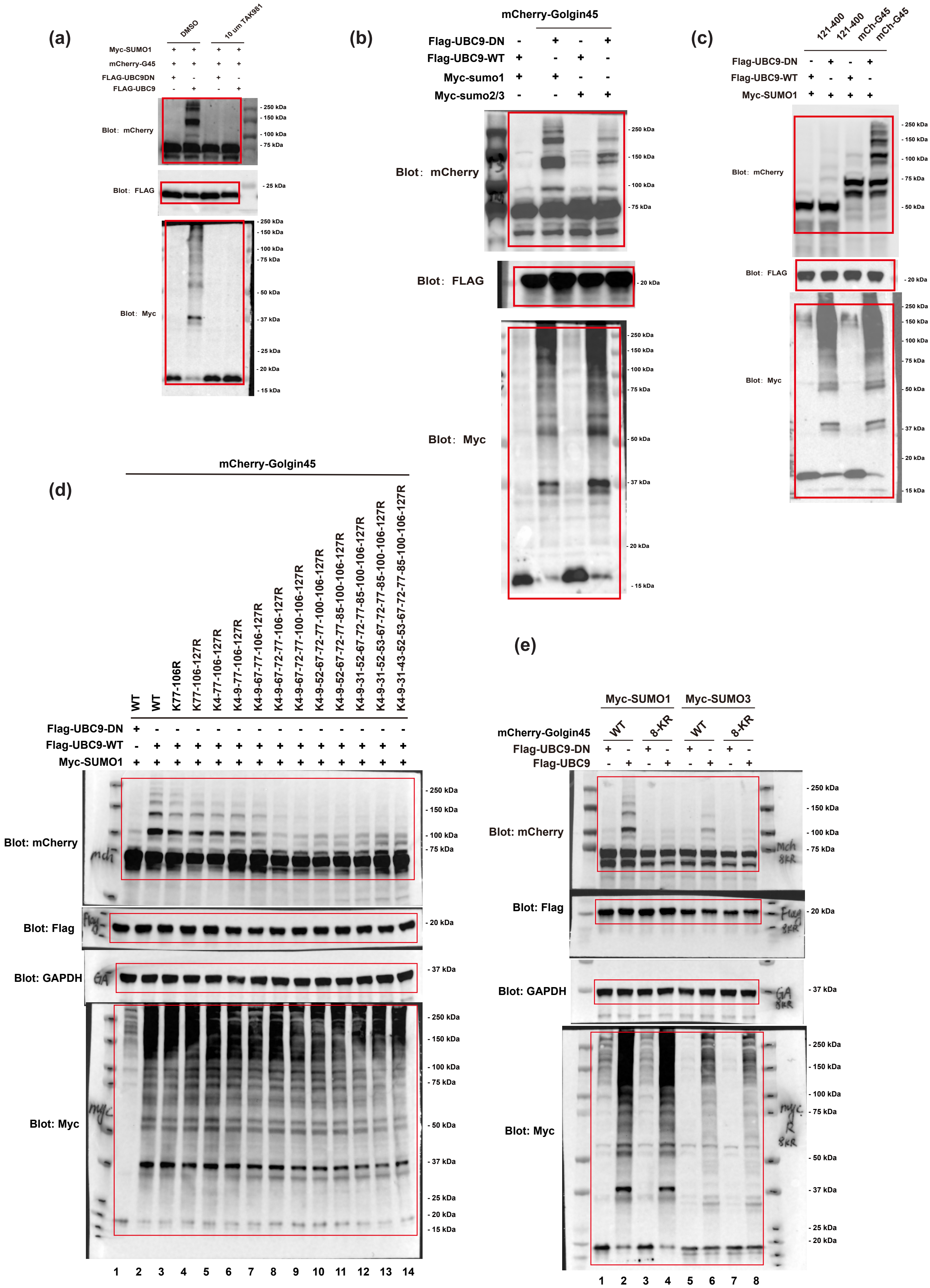

Unprocessed blots of Figure 1

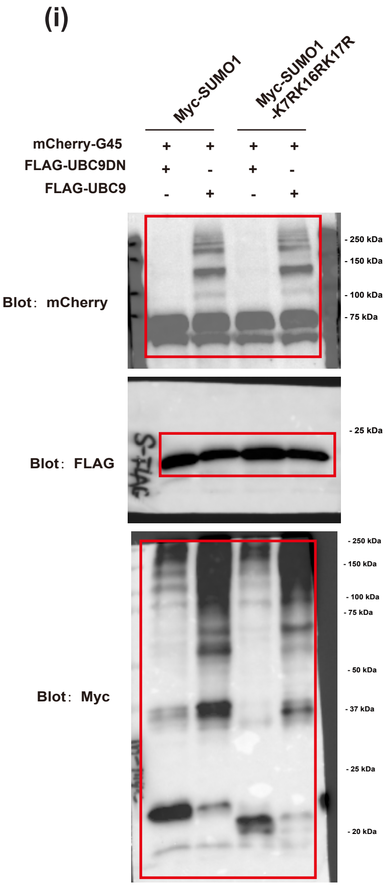

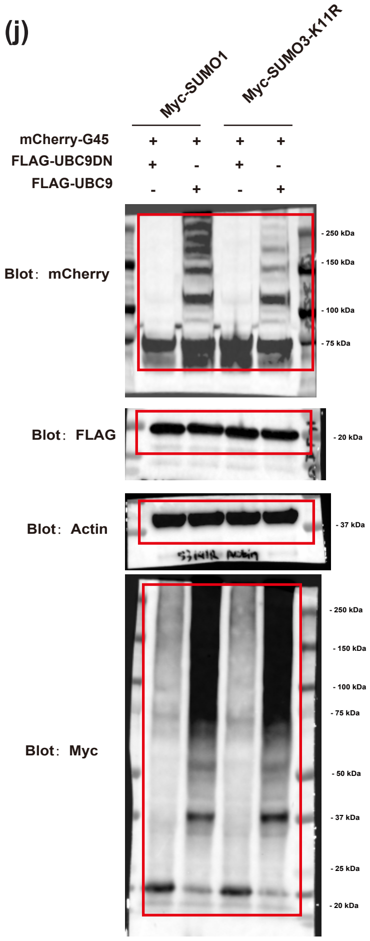

Unprocessed blots of Figure 2

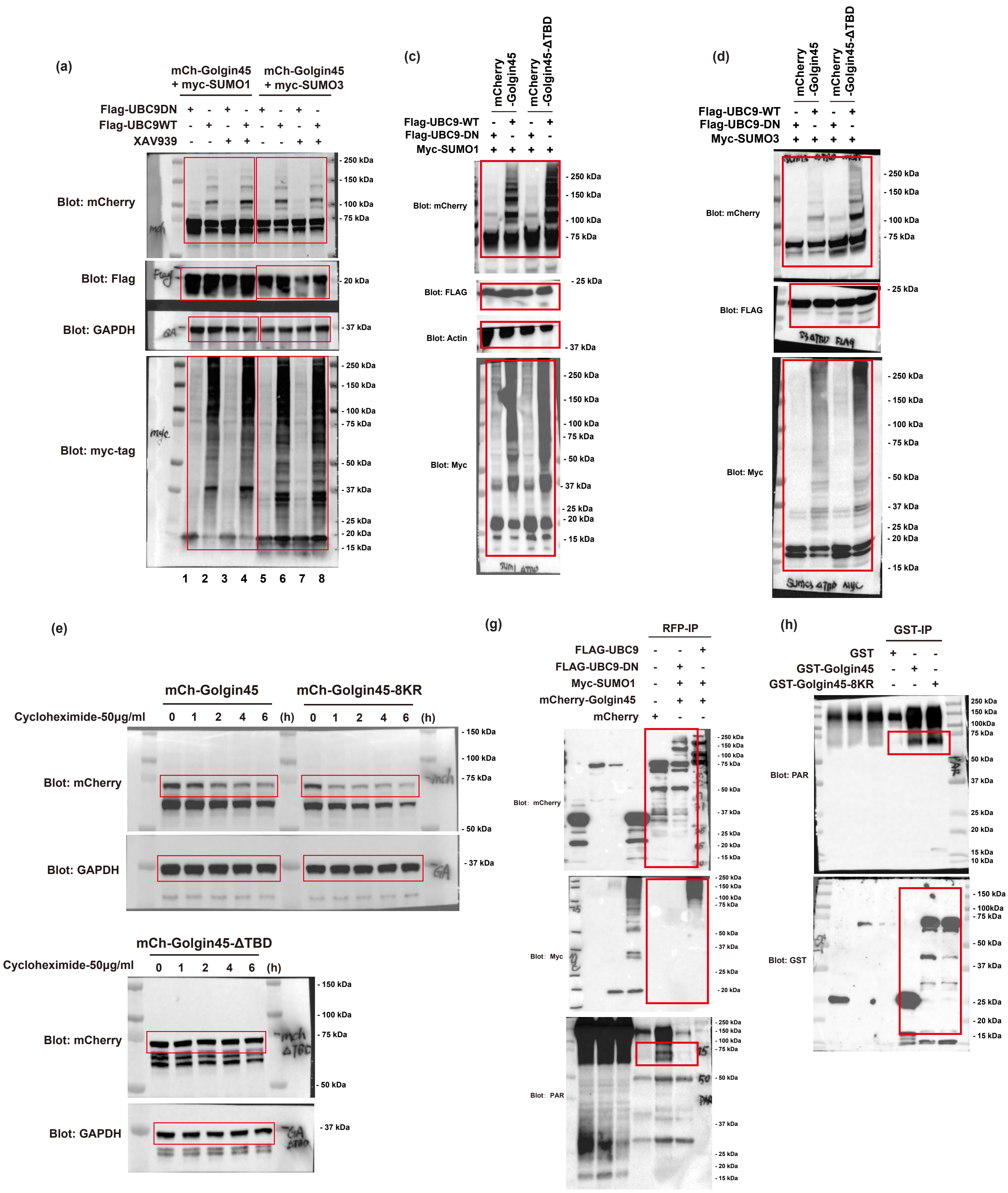

Unprocessed blots of Figure 3

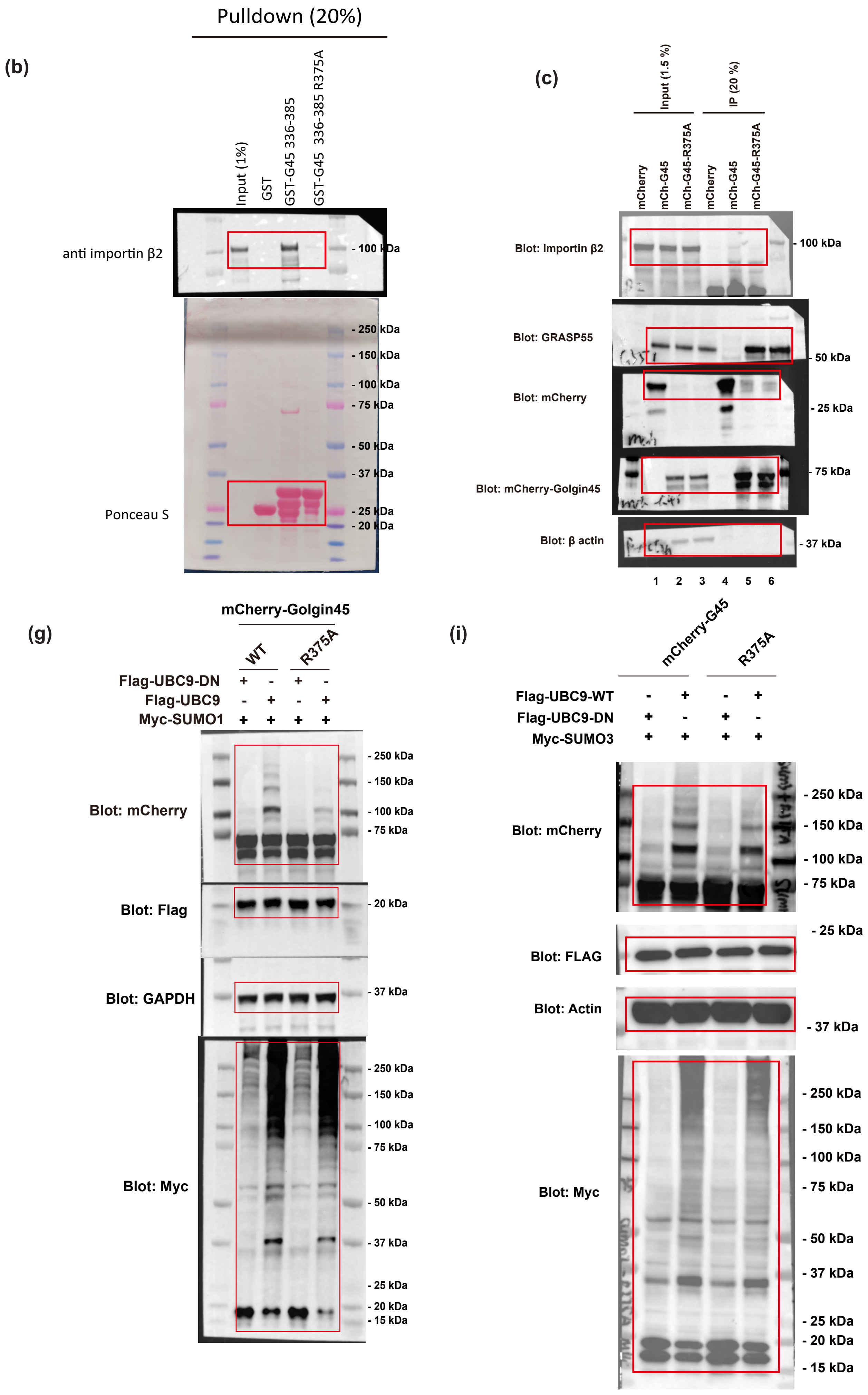

Unprocessed blots of Figure 4

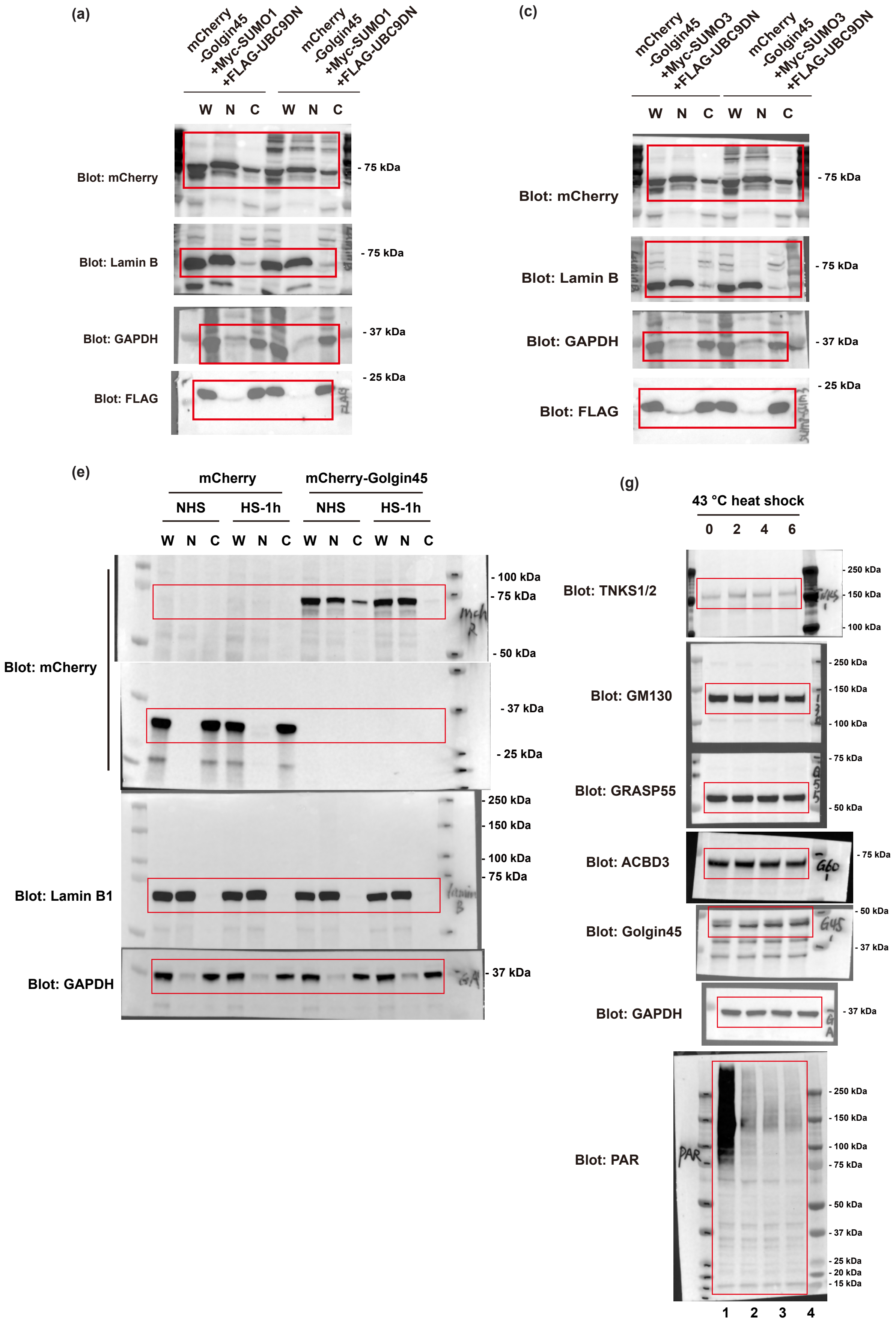

Unprocessed blots of Figure 4

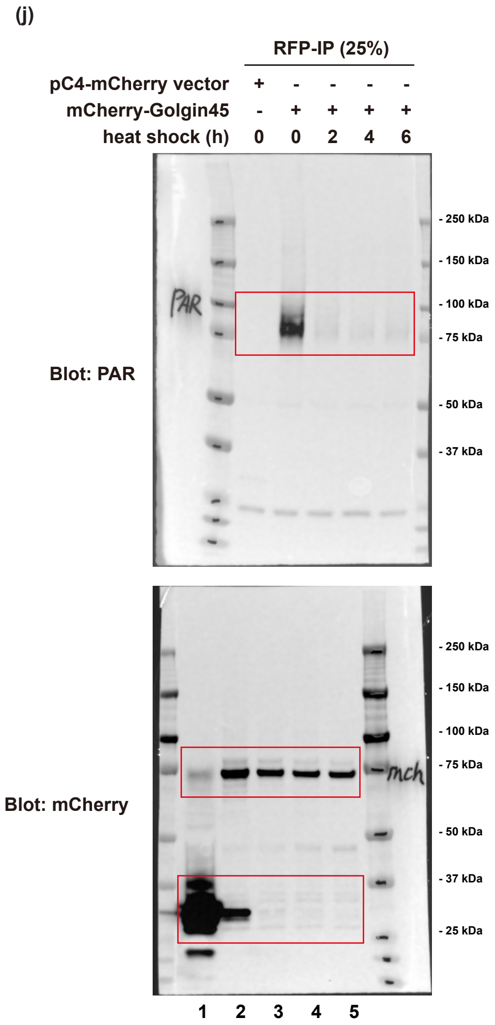

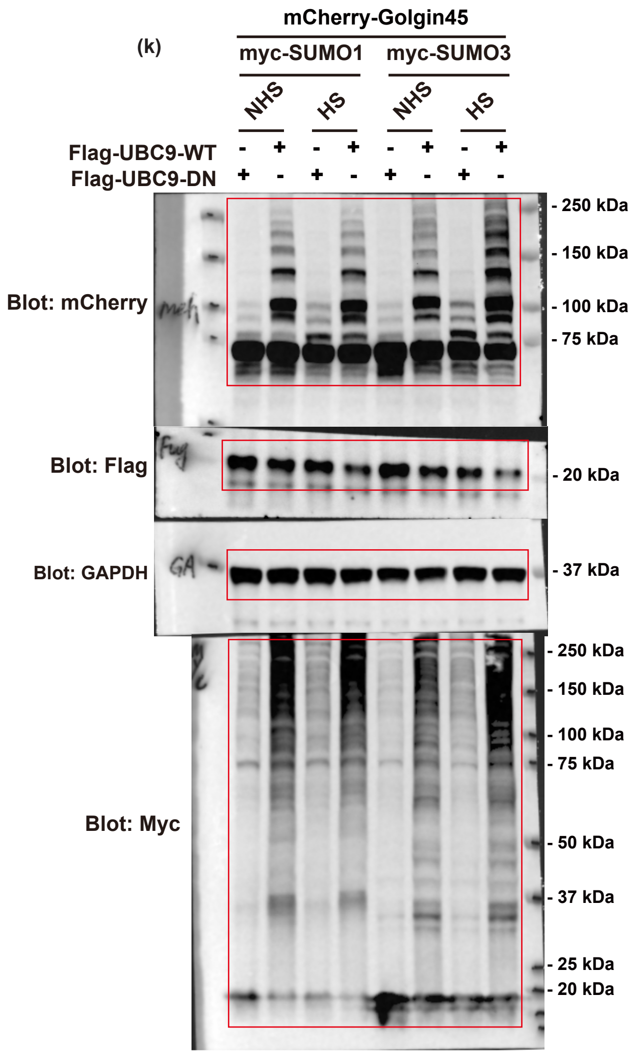

Unprocessed blots of Figure 5

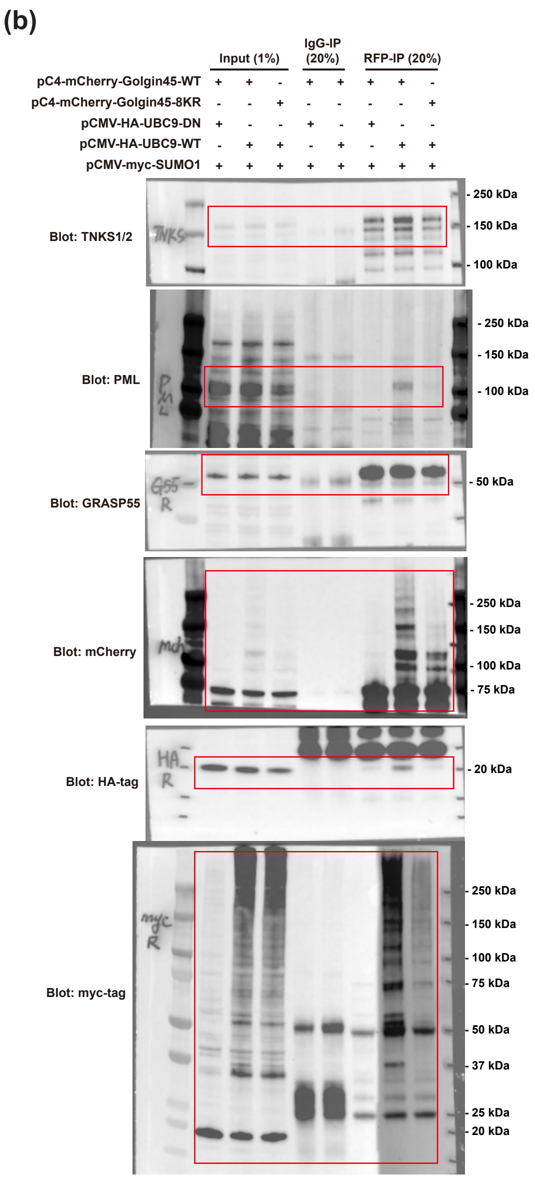

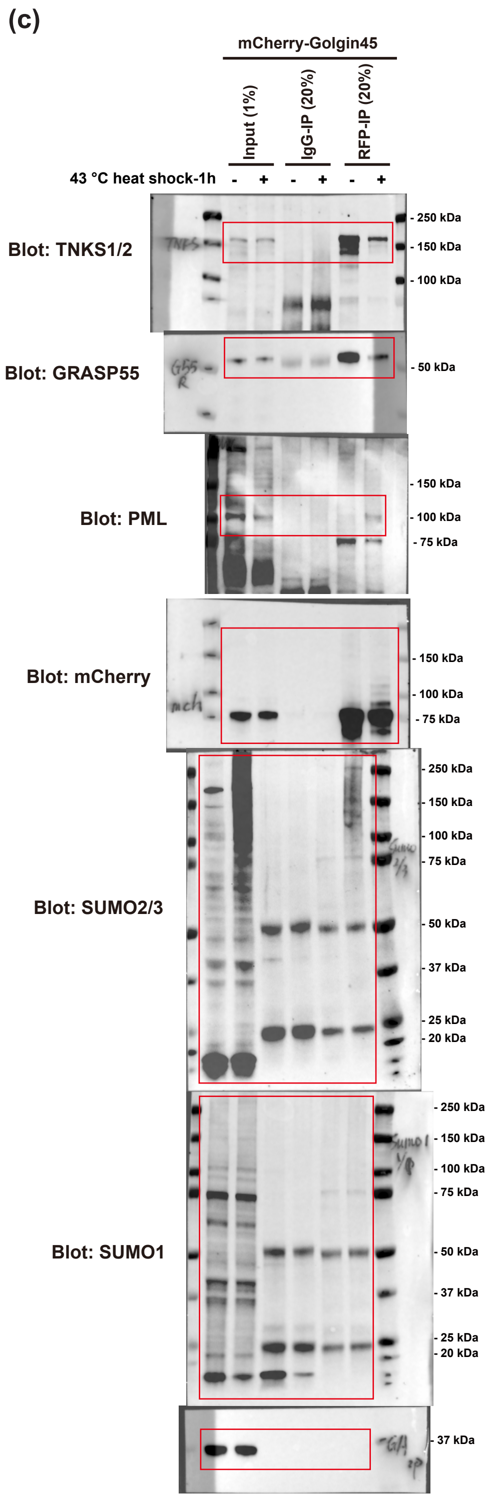

Unprocessed blots of Figure 6

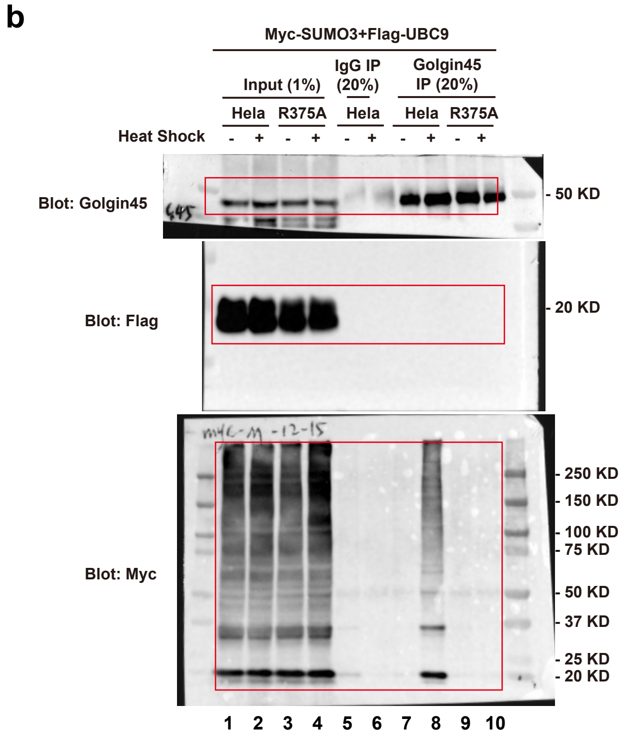

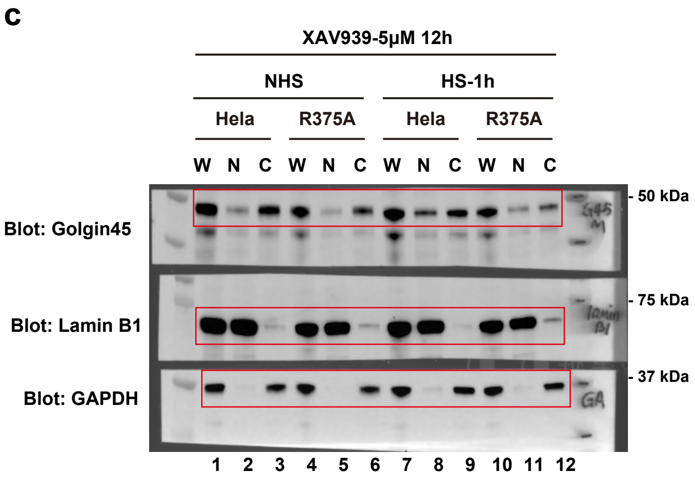

Supplementary Figure 1

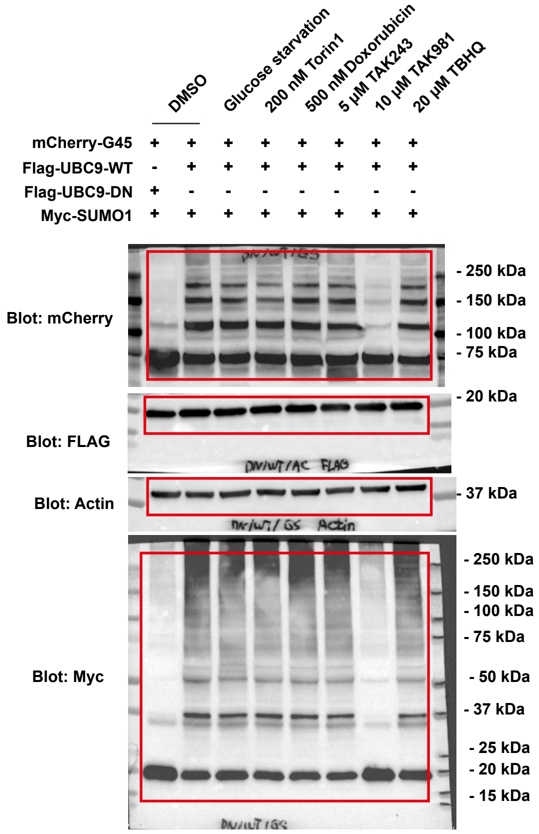

Supplement: Supplementary file 1 — Supplementary information [file 42003_2024_6232_MOESM1_ESM.pdf]
