# Supplementary material for: SUMOylated Golgin45 associates with PML-NB to transcriptionally regulate lipid metabolism genes during heat shock stress
Source: Commun Biol. 2024 May 6;7:532. doi: 10.1038/s42003-024-06232-3 (PMC11074300; doi:10.1038/s42003-024-06232-3)
Supplement: Supplementary file 2 — Description of Supplementary Materials [file 42003_2024_6232_MOESM2_ESM.docx]

**Description of Additional Supplementary Files**

**File name**: Supplementary Data 1

**Description:** SUMOylated Golgin45 CoIP-MS results.

**File name**: Supplementary Data 2

**Description:** RNA-seq data of HeLa-WT vs HeLa-WT-HS.

**File name:** Supplementary Data 3

**Description:** RNA-seq data of HeLa-WT-HS vs HeLa-Golgin45-KO-HS.

**File name:** Supplementary Data 4

**Description:** The source data for the graphs in the paper
