# Supplementary material for: SUMOylated Golgin45 associates with PML-NB to transcriptionally regulate lipid metabolism genes during heat shock stress
Source: Commun Biol. 2024 May 6;7:532. doi: 10.1038/s42003-024-06232-3 (PMC11074300; doi:10.1038/s42003-024-06232-3)
Supplement: Supplementary file 7 — reporting summary [file 42003_2024_6232_MOESM7_ESM.pdf]

nature portfolio

Corresponding author(s): Yi Qian, Xihua Yue and Intaek Lee

Last updated by author(s): Apr 9, 2024

Reporting Summary

Nature Portfolio wishes to improve the reproducibility of the work that we publish. This form provides structure for consistency and transparency in reporting. For further information on Nature Portfolio policies, see our Editorial Policies and the Editorial Policy Checklist.

Statistics

For all statistical analyses, confirm that the following items are present in the figure legend, table legend, main text, or Methods section.

n/a	Confirmed	
☐	☒	The exact sample size (*n*) for each experimental group/condition, given as a discrete number and unit of measurement
☐	☒	A statement on whether measurements were taken from distinct samples or whether the same sample was measured repeatedly
☐	☒	The statistical test(s) used AND whether they are one- or two-sided<br>*Only common tests should be described solely by name; describe more complex techniques in the Methods section.*
☐	☒	A description of all covariates tested
☐	☒	A description of any assumptions or corrections, such as tests of normality and adjustment for multiple comparisons
☐	☒	A full description of the statistical parameters including central tendency (e.g. means) or other basic estimates (e.g. regression coefficient) AND variation (e.g. standard deviation) or associated estimates of uncertainty (e.g. confidence intervals)
☐	☒	For null hypothesis testing, the test statistic (e.g. *F*, *t*, *r*) with confidence intervals, effect sizes, degrees of freedom and *P* value noted<br>*Give P values as exact values whenever suitable.*
☒	☐	For Bayesian analysis, information on the choice of priors and Markov chain Monte Carlo settings
☒	☐	For hierarchical and complex designs, identification of the appropriate level for tests and full reporting of outcomes
☐	☒	Estimates of effect sizes (e.g. Cohen's *d*, Pearson's *r*), indicating how they were calculated

Our web collection on statistics for biologists contains articles on many of the points above.

Software and code

Policy information about availability of computer code

Data collection	ZEN software (Carl Zeiss), Image Lab software (Bio-Rad).
Data analysis	Prism 9.0 (Graphpad), Fiji (ImageJ).

For manuscripts utilizing custom algorithms or software that are central to the research but not yet described in published literature, software must be made available to editors and reviewers. We strongly encourage code deposition in a community repository (e.g. GitHub). See the Nature Portfolio guidelines for submitting code & software for further information.

Data

Policy information about availability of data

All manuscripts must include a data availability statement. This statement should provide the following information, where applicable:

- Accession codes, unique identifiers, or web links for publicly available datasets
- A description of any restrictions on data availability
- For clinical datasets or third party data, please ensure that the statement adheres to our policy

The mass spectrometry proteomics data have been deposited to the ProteomeXchange Consortium via the PRIDE partner repository with the dataset identifier PXD051237. The RNA-Seq data presented in this study are available in the NCBI Sequence Read Archive (NCBI-SRA BioProject ID: PRJNA1097982). All materials and data supporting this study are available from the corresponding authors (yuexh@shanghaitech.edu; qianyi@shanghaitech.edu; intalee@gmail.com) upon reasonable request. Images of uncropped blots are provided in Supplementary Fig. 4. Source data for graphs are available in Supplementary Data 4.

Human research participants

Policy information about studies involving human research participants and Sex and Gender in Research.

Reporting on sex and gender	Not applicable.
Population characteristics	Not applicable.
Recruitment	Not applicable.
Ethics oversight	Not applicable.

Note that full information on the approval of the study protocol must also be provided in the manuscript.

Field-specific reporting

Please select the one below that is the best fit for your research. If you are not sure, read the appropriate sections before making your selection.

☒ Life sciences ☐ Behavioural & social sciences ☐ Ecological, evolutionary & environmental sciences

For a reference copy of the document with all sections, see nature.com/documents/nr-reporting-summary-flat.pdf

Life sciences study design

All studies must disclose on these points even when the disclosure is negative.

Sample size	Sample size were chosen large enough to account for the heterogeneity among cells. Relevant literature study and search guided us to determine the group numbers in each experiments. For quantification of immunofluorescence data, at least 30 different cells in each experiments were included for data analysis. At least three independent experiments were performed to obtained data and to determine the statistical significance.
Data exclusions	No data were excluded from the study.
Replication	All attempts for replication were successful. At least three independent applications were carried out for each qualification analysis.
Randomization	Samples were picked randomly to statistical analysis.
Blinding	No animals or human research participants are involved in this study.

Reporting for specific materials, systems and methods

We require information from authors about some types of materials, experimental systems and methods used in many studies. Here, indicate whether each material, system or method listed is relevant to your study. If you are not sure if a list item applies to your research, read the appropriate section before selecting a response.

Materials & experimental systems

n/a	Involved in the study	
☐	☒	Antibodies
☐	☒	Eukaryotic cell lines
☒	☐	Palaeontology and archaeology
☒	☐	Animals and other organisms
☒	☐	Clinical data
☒	☐	Dual use research of concern

Methods

n/a	Involved in the study	
☒	☐	ChIP-seq
☒	☐	Flow cytometry
☒	☐	MRI-based neuroimaging

Antibodies

Antibodies used	The following antibodies were used : rabbit polyclonal anti-PML (21041-1-AP, proteintech, 1:1000 for WB), mouse monoclonal anti-PML (ab96051, Abcam, 1:500 for IF), rabbit polyclonal anti-mCherry (ab167453, Abcam, 1:2000 for WB), anti-GM130 (1:2000 for WB and 1:1000 for IF, ab52649, Abcam), mouse monoclonal anti-FLAG (F1804, Sigma-Aldrich, 1:2000 for WB), rabbit anti-Myc (2278S, Cell Signaling Technology, 1:2000 for WB), mouse anti-Actin (3700s, Cell Signaling Technology, 1:5000 for WB), anti-GST (RPN1236, Cytiva, 1:2000 for WB), rabbit polyclonal anti-Mono-ADP Ribose (83732S, Cell Signaling Technology, 1:1000 for WB), rabbit polyclonal

	anti-Golgin45 (PA5-30714, Thermo, 1:1000 for WB), anti-ACBD3 (HPA015594, Sigma Aldrich, 1:2000 for WB), mouse monoclonal anti-GRASP55 (ab211532, Abcam, 1:1000 for WB), anti-GAPDH (KC-5G5, Kangchen Bio-tech, 1:5000 for WB), mouse monoclonal anti-Transportin 1 (ab10303, Abcam, 1:2000 for WB), rabbit polyclonal anti-Lamin B (12987-1-AP, Proteintech, 1:1000 for WB), mouse monoclonal anti-Tankyrase1/2 (sc-365897, Santa Cruz, 1:1000 for WB), mouse anti-HA-Tag (2367s, Cell Signaling Technology, 1:1000 for WB). Anti-Rabbit Alexa Fluor 488 (A21441, 1:500), Alexa Fluor 568 (A10042, 1:500), Alexa Fluor 647 (A21245, 1:500) and anti-Mouse Alexa Fluor 488 (A21200, 1:500), Alexa Fluor 568 (A10037, 1:500), Alexa Fluor 647 (A21236, 1:500) for Immunofluorescence were obtained from ThermoFisher.
Validation	Commercial antibodies have been verified by the manufacturers as shown on their website. All antibodies were used based on manufacturer recommendations.

Eukaryotic cell lines

Policy information about cell lines and Sex and Gender in Research

Cell line source(s)	HeLa cell line was purchased from the American Type Culture Collection.
Authentication	HeLa cells were authenticated by STR profiling.
Mycoplasma contamination	All cell lines were routinely tested for the mycoplasma contamination and were negative.
Commonly misidentified lines (See ICLAC register)	None of the cell lines used in this study is listed in the database of commonly misidentified cell kines maintained by iCLAC.
